# Cellular responses at the application site of a high-density microarray patch delivering an influenza vaccine in a randomized, controlled phase I clinical trial

**DOI:** 10.1371/journal.pone.0255282

**Published:** 2021-07-30

**Authors:** Alexandra C. I. Depelsenaire, Katey Witham, Margaret Veitch, James W. Wells, Christopher D. Anderson, Jason D. Lickliter, Steve Rockman, Jesse Bodle, Peter Treasure, Julian Hickling, Germain J. P. Fernando, Angus H. Forster

**Affiliations:** 1 Vaxxas Pty Ltd, Brisbane, Queensland, Australia; 2 The University of Queensland Diamantina Institute, Woolloongabba, Queensland, Australia; 3 Department of Clinical and Experimental Medicine, Linkoping University, Linköping, Sweden; 4 Nucleus Network Pty Ltd, Melbourne, Victoria Australia; 5 Seqirus Pty Ltd, Parkville, Victoria, Australia; 6 Department of Microbiology and Immunology, University of Melbourne, at The Peter Doherty Institute for Infection and Immunity, Melbourne, Victoria, Australia; 7 Peter Treasure Statistical Services Ltd, Kings Lynn, United Kingdom; 8 Working in Tandem Ltd, Cambridge, United Kingdom; 9 The University of Queensland, School of Chemistry & Molecular Biosciences, Faculty of Science, Brisbane, Queensland, Australia; IAVI, UNITED STATES

## Abstract

Microarray patches (MAPs) have the potential to be a safer, more acceptable, easier to use and more cost-effective method for administration of vaccines when compared to the needle and syringe. Since MAPs deliver vaccine to the dermis and epidermis, a degree of local immune response at the site of application is expected. In a phase 1 clinical trial (ACTRN 12618000112268), the Vaxxas high-density MAP (HD-MAP) was used to deliver a monovalent, split inactivated influenza virus vaccine into the skin. HD-MAP immunisation led to significantly enhanced humoral responses on day 8, 22 and 61 compared with IM injection of a quadrivalent commercial seasonal influenza vaccine (Afluria Quadrivalent®). Here, the aim was to analyse cellular responses to HD-MAPs in the skin of trial subjects, using flow cytometry and immunohistochemistry. HD-MAPs were coated with a split inactivated influenza virus vaccine (A/Singapore/GP1908/2015 [H1N1]), to deliver 5 μg haemagglutinin (HA) per HD-MAP. Three HD-MAPs were applied to the volar forearm (FA) of five healthy volunteers (to achieve the required 15 μg HA dose), whilst five control subjects received three uncoated HD-MAPs (placebo). Local skin response was recorded for over 61 days and haemagglutination inhibition antibody titres (HAI) were assessed on days 1, 4, 8, 22, and 61. Skin biopsies were taken before (day 1), and three days after HD-MAP application (day 4) and analysed by flow-cytometry and immunohistochemistry to compare local immune subset infiltration. HD-MAP vaccination with 15 μg HA resulted in significant HAI antibody titres compared to the placebo group. Application of uncoated placebo HD-MAPs resulted in mild erythema and oedema in most subjects, that resolved by day 4 in 80% of subjects. Active, HA-coated HD-MAP application resulted in stronger erythema responses on day 4, which resolved between days 22–61. Overall, these erythema responses were accompanied by an influx of immune cells in all subjects. Increased cell infiltration of CD3^+^, CD4^+^, CD8^+^ T cells as well as myeloid CD11b^+^ CD11c^+^ and non-myeloid CD11b^-^ dendritic cells were observed in all subjects, but more pronounced in active HD-MAP groups. In contrast, CD19^+^/CD20^+^ B cell counts remained unchanged. Key limitations include the use of an influenza vaccine, to which the subjects may have had previous exposure. Different results might have been obtained with HD-MAPs inducing a primary immune response. In conclusion, influenza vaccine administered to the forearm (FA) using the HD-MAP was well-tolerated and induced a mild to moderate skin response with lymphocytic infiltrate at the site of application.

## Introduction

Whilst conventional immunisation routes such as intramuscular (IM), subcutaneous (SC), or intradermal (ID) injection are the standard, dermal immunisation by microneedle array patches (MAPs) offers an alternative [1]. Dermal routes take advantage of the skin’s high abundance of antigen presenting cells (APCs) and have been reported in humans to require only a fraction of the antigen dose needed by IM or SC injection to achieve protective responses [2–8]. This is believed to be due to the ability of dermal immunisations to deliver the antigen directly into the vicinity of APCs such as dermal dendritic cells (dDCs), and the local network of draining lymphatic vessels carrying antigen to the draining lymph nodes (dLN) [9].

MAPs have successfully delivered therapeutics, including vaccines, into the upper dermis as reported in several pre-clinical [10, 11] and clinical studies [12–14]. Pre-clinical studies have also demonstrated the dose-sparing potential of MAPs [15, 16]. Initial clinical trials found MAPs to be safe, well tolerated and preferred by recipients to needle and syringe [17, 18]. Furthermore, MAPs can induce immune responses at least as potent as IM injections [12–14, 19]. Moreover, all tested MAPs have resulted in transient erythema, irrespective of the influenza antigen, excipients used, or MAP format (e.g. solid micro-projections or dissolving MAPs) [12–14, 17, 19]. In studies with solid micro-projection MAPs, erythema was apparent within 10 min post application of high-density (HD) silicon Nanopatch (NP) [12, 17] or HD-MAPs [19], peaking between day 2 and 4 post HD-MAP [13, 19] or NP application [12]. In most cases, erythema resolved by day 3 post application of uncoated MAPs and day 7 post application of excipient coated MAPs [17], and between day 7 and day 25 post application of influenza antigen coated MAPs [12, 19]. In studies with dissolving MAPs (delivering influenza antigen), Rouphael *et al* reported erythema at day 7 with resolution at 28 days [14], whereas Hirobe *et al* showed erythema over 21 days, peaking on day 2 post application [13]. To our knowledge, no clinical study to date has assessed the cellular responses *in situ* at the application sites following vaccine delivery by MAPs, presenting a significant gap in the scientific literature of local skin responses to MAP vaccination.

We have conducted a first-in-human study with solid HD-MAPs coated with inactivated split influenza virus haemagglutinin (HA) from A/Singapore/GP1908/2018 (A/Michigan/45/2015(H1N1)-like, (A/Sing)) vaccine (ACTRN 12618000112268). The study was a two-part randomised, partially double-blind, placebo-controlled study in healthy subjects, with 4 parallel treatment groups in Part A and 9 parallel treatment groups in Part B [19]. The primary objective of the clinical study was to evaluate safety and tolerability of the A/Sing-coated (active) HD-MAP compared with uncoated (placebo) HD-MAP, A/Sing antigen delivered IM, as well as commercially available quadrivalent seasonal influenza vaccine (QIV) delivered IM. This study also demonstrated for the first-time in humans, dose-sparing using HD-MAPs to deliver a vaccine compared with IM injection [19]. Several exploratory objectives (antibody-dependent cellular cytotoxicity, CD4^+^ T cell cytokine production, memory B cell activation, recognition of non-vaccine strains and local skin responses) were included [19] to provide information about the immune responses to MAP vaccination compared to the current IM vaccination route as well as to understand local skin responses. Here, we present and discuss the findings from two subgroups (receiving active or placebo HD-MAPS) of Part B of the study, where punch biopsies were taken pre-HD-MAP application (day 1) and 3 days post-HD-MAP application (day 4) from within the HD-MAP application site. Analysis by flow cytometry aimed at broadly screening for immune cell types present in the biopsies provided a quantitative measurement of T cells, B cells and DCs and an indication of the types of immune activity occurring at the HD-MAP application site. Spatial and visual assessment of the biopsies included H&E stained sections as well as immunofluorescent (IF) marker analyses for T cells, B cells, and dendritic cells (DCs).

## Methods

### Trial subjects and study design

The study was approved by the Bellberry Human Research Ethics Committee (South Australia, Australia), and conducted in accordance with the Australian National Health and Medical Research Council’s National Statement of Ethical Conduct in Human Research (2007; incorporating all updates as at May 2015). Written informed consent was obtained from all participants. The trial was registered with Australian New Zealand Clinical Trials Registry (ANZCTR.org.au), trial ID ACTRN12618000112268/U1111-1207-3550 on 25 January 2018, and recruitment started 09 March 2018.

**Fig 1:**
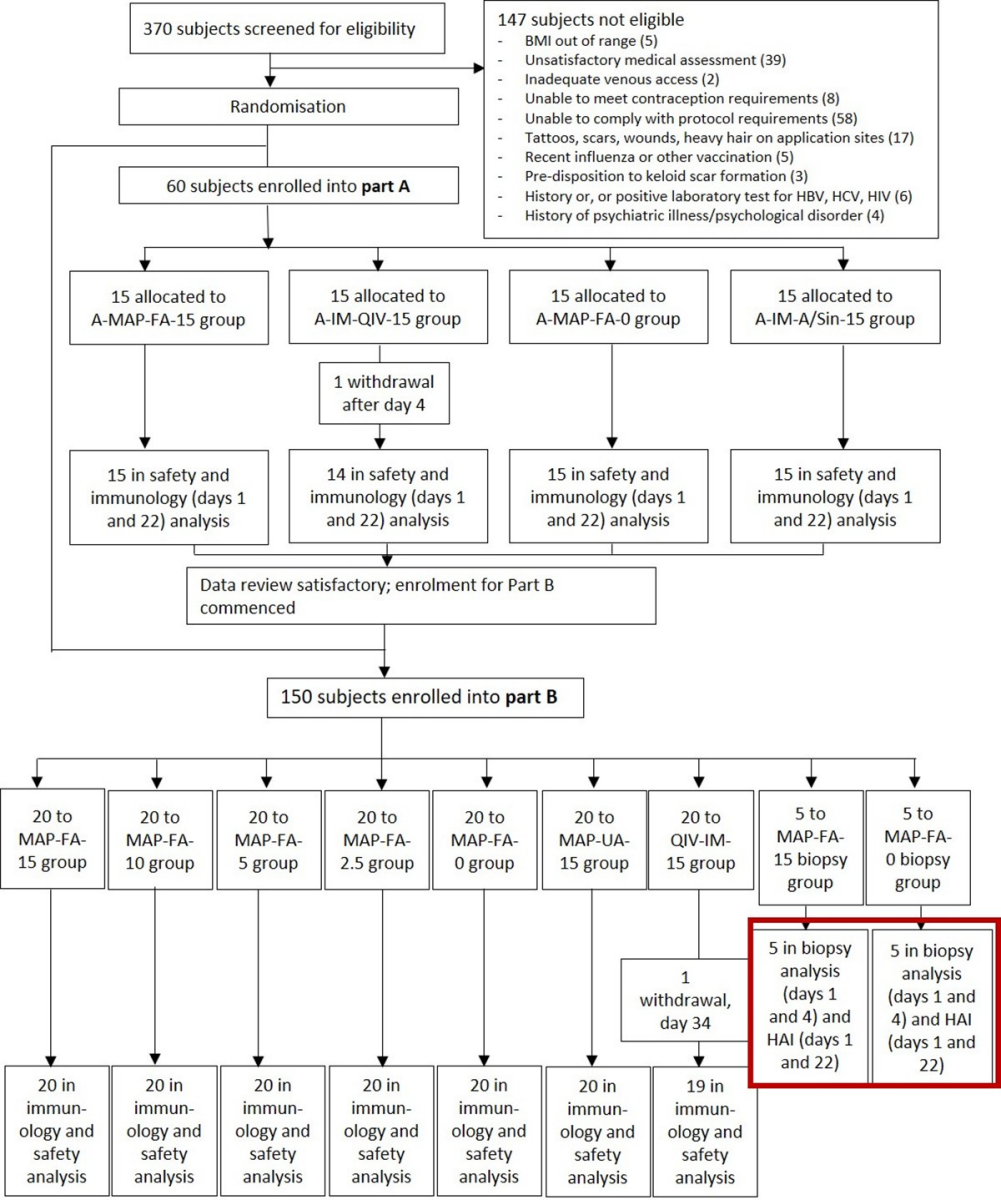
Clinical trial profile. The two groups providing biopsies for analysis are highlighted in the red box.

A two-part randomised, partially-blinded, placebo-controlled trial study was conducted at Nucleus Network Pty Ltd (Melbourne, VIC) (refer to [19] for details). Fig 1 shows the two biopsy groups (red box) included in the trial profile that were the subjects of the analysis reported here. Due to the objective of this study being an exploratory assay, the sample size was not based on any formal statistical calculations. Randomisation was pre-determined, and sealed subject-specific code break envelopes were prepared by the statistician responsible for preparing the randomisation. Subjects and clinical were blind as to which MAP treatment was administered. All laboratory personnel were blind to treatment and subject allocation. Healthy males and females (non-pregnant and non-nursing) aged 18–50 years, with a BMI in the range of 18–30 kg/m^2^ were randomly allocated into one of nine vaccination groups for part B (Fig 1). Ten subjects (n = 5 per group) were randomised into placebo (i.e. uncoated) or active (i.e. 15 μg A/Sing vaccine-coated) HD-MAP groups. The enrolled subjects were not selected based on HAI titres. The demographics of the two groups are shown in Table 1. Three days post application (day 4) was chosen as an appropriate timepoint to take biopsies and investigate immune cell profile in the skin in response to MAP application based on earlier observations of skin responses to MAP vaccination [12]. Serological and cellular immune responses from skin biopsies were assessed pre- and post-vaccination.

**Table 1:** Subject demographics of the skin biopsy groups (n = 10).

	Active	Placebo
**Number**	5	5
**Age:** mean (SD)	39.6 (8.1)	29.6 (4.9)
**Age:** range (years)	31–50	24–37
**Sex:** female; number (%)	4 (80)	3 (60)
**Sex:** male; number (%)	1 (20)	2 (40)
**BMI** (kg/m^2^): mean (SD)	24.72 (0.8)	22.72 (2.61)
**Race: white;** number (%)	5 (100)	5 (100)
**Ethnicity**: Non-Hispanic and Non-Latino; number (%)	4 (80)	4 (80)
**Ethnicity**: Hispanic or Latino; number (%)	1 (20)	1 (20)

### Study product

Egg-based split influenza A/Singapore/GP1908/2015 (A/Michigan/45/2015 [H1N1-like]) MPH (A/Sing) was supplied by Seqirus Pty Ltd (Australia). Sulfobutyl ether (β) cyclodextrin (SBECD) (Captisol® Ligand, USA), was added at a ratio of 4 to 1 (% w/w) HA protein to preserve antigen potency during coating and storage. MAPs were prepared uncoated and gamma-sterilised for use in placebo groups or coated with split influenza A/Sing MPH between 05 Feb 2018 and 27 Mar 2018 and stored at 2–8°C until used (08 May 2018) as described elsewhere [19]. MAPs coated with A/Sing HA antigen coated at 5 μg per HD-MAP were thermostable when stored at 2 to 8°C, 25°C, or 40°C for at least 12 months [19].

### Vaccination procedure

For full details, refer to [19]. Briefly, subjects had three solid HD-MAPs applied using a hand-held single-use high-velocity spring-loaded applicator to the volar forearm and kept in place for 2 minutes. Upon HD-MAP application and projections inserted into the skin, active and placebo coating then dissolve off the projections into the epidermis and upper dermis. Subjects in the active group received three A-Sing-coated HD-MAPs; each HD-MAP delivered 5 μg of HA. Subjects in the placebo group received three uncoated HD-MAPs. Subjects were monitored by clinic safety assessment visits at days 1, 2, 4, 8, 22, and 61; and phone calls at days 3, 36 and 50. On day 1, all vaccination sites were assessed at pre-vaccination, 10 minutes, 1 and 2 hours after HD-MAP or IM administration. Photographs of the treatment sites were taken at every in-clinic review (S1 Fig). Skin responses were assessed for erythema and oedema, with scores combined to generate a Skin Irritation Index (SII) as described elsewhere [12, 17].

### Clinical biopsy samples

Each subject had two 4 mm punch biopsies (ProSciTech, Australia) taken from their volar forearm at each of the two time-points, one for histological and one for flow cytometric analyses. The day 1 biopsies were taken prior to HD-MAP application, from the opposite arm to the one used for HD-MAP application. On day 4, two biopsies were taken from each subject, each from within a different HD-MAP application site, referred to as sites 1 and 3 (site 2 was used for monitoring undisturbed skin reactions). The biopsy sites were stitched with dissolving suture material following removal of the biopsies.

The skin biopsies for flow cytometry were shipped at 2–8°C, overnight in 1 mL RPMI1640/10% heat inactivated foetal bovine serum (HI FCS) shipping medium supplemented with antibiotics (Pen-Strep) from Nucleus Network (Melbourne, Australia) to Vaxxas Pty Ltd (Brisbane, Australia). The skin biopsies for histology were shipped overnight under ambient conditions in 10% neutral buffered formalin (NBF) from Nucleus Network (Melbourne, Australia) to QIMR (Brisbane, Australia) for further sample processing. See S1 Methods for more information.

### Flow cytometry

Upon receipt at Vaxxas Pty Ltd, the skin biopsies were digested immediately to release individual cells for staining and analysis by flow cytometry using a protocol adapted from [20], to maximise cell viability and cell yield following overnight transportation. Specifically, subcutaneous fat was removed, biopsies were transferred into 1 mL RPMI1640/2% HI FCS containing 3 mg/mL Collagenase P (*Clostridium histolyticum*, Roche Life Science, Australia) and 5 μg/mL DNAse (Roche Life Science, Australia), cut into pieces (0.5 × 0.5 mm) and incubated for 60 min at 37°C with continuous agitation (plate shaker 400 rpm). The cell suspension was pushed through a 70 μm strainer, collected with the shipping medium and diluted to 10 mL with PBS/2% HI FCS/2mM EDTA. After centrifugation (300xg, 5 min, 4°C), the cell pellet was resuspended and transferred through a 40 μm cell strainer and collected in 5 mL FACS tubes in a final volume of 3 mL with Dulbecco’s Phosphate Buffered Saline (DPBS) and re-centrifuged. Following this wash step, the cells were resuspended in DPBS to a final volume of 100 μL. Once isolated, cells were stained with 14 fluorescent antibodies. Refer to S1 Methods and S1 Table for the summary of methodology and fluorescent antibodies used. The gating strategy is shown in S2 Fig.

### Immunohistochemistry (immunofluorescence)

Upon excision, biopsies were fixed for 24 hours in 10% NBF, followed by ethanol dehydration and paraffin embedding. All ten biopsy samples were then processed into one tissue matrix array (TMA), and sectioned at 5 μm thickness on a Thermo Fisher Shandon Finesse 325 microtome. Sections were then either stained with Haematoxylin and Eosin (H&E; Haematoxylin, Sigma-Aldrich, USA; Eosin Y, ProScitech, Australia), or for IF. Eleven primary antibodies and appropriate secondary antibodies conjugated to AF555 were used (S1 Methods and S2 Table).

### Statistical analysis

As described in the main publication [19], the sample sizes for the Phase I clinical study were not based on formal statistical calculations, and in particular the sample size for both biopsy subgroups was small (n = 5 per group) due to the invasive nature of sample collection. Mean and standard deviation for age and BMI was reported along with the age range for biopsy subject demographics.

For antibody responses in biopsy subjects, geometric mean of the HAI titres (GMT) as well as the 95% confidence interval (95% CI) of the geometric mean were determined. Seroconversion rates were defined as percentage of participants with an HAI titre ≥1:40 post-vaccination for initially seronegative (HAI <10 at baseline) participants, or ≥4 fold the pre-vaccination antibody titre for initially seropositive (HAI ≥10 at baseline) participants; seroprotection rates were defined as percentage of participants with HAI titre ≥1:40.

For all immune cell analyses presented here, two-way ANOVA with was performed with matched subjects across time points. For flow cytometry, the frequency of stained cells was assessed. For immunohistochemistry, the sum of the marker frequencies from each subject were combined for assessment. The model included terms for time, treatment and their interaction. Quantiles of the residuals were plotted against the quantiles for a normal distribution (normal probability or QQ plot) and indicated that the assumption of normality is reasonable using this approach. Residual and homoscedascity plots were assessed when selecting this methodology. As a result, it was decided to be appropriate to log transform the immunofluorescent cell infiltration data (Fig 9) because the standard deviations were approximately proportional to the mean.

The two-way ANOVA was used to determine if there were statistically significant differences in the presence of immune cells in biopsy samples in response to both treatment (active and placebo) and time (day 1 and day 4). Treatments within the same timepoints were compared. Comparisons were made between subjects in active and placebo groups, both on day 1 and day 4; as well as between day 1 and day 4 within the active and placebo groups. Statistical significance was declared at the p < 0.05 level. No adjustment for multiplicity was made as these were exploratory analyses of Phase I data, reported P-values should be interpreted with caution.

Analyses were performed with GraphPad PRISM® (version 9.1.0; La Jolla, San Diego, CA, USA).”

## Results

### Subjects and study procedures

For part B, ten of the 150 subjects were enrolled between 20 April 2018 and 26 June 2018. Details of the design and results from the full phase I clinical trial consisting of 210 subjects are provided elsewhere [19]. Within this study, two subgroups (five subjects per group) receiving three active 5 μg HD-MAPs (totalling 15 μg) or three placebo HD-MAPs, were included to assess local skin responses to HD-MAPs. Biopsies were taken pre-HD-MAP application (day 1) and 3 days post-HD-MAP application (day 4). There were no obvious differences in demographics between the two groups (Table 1).

### Skin and tolerability assessment

In total, 5 of 5 subjects in the active (HA-coated) HD-MAP group reported treatment-emergent adverse events (TEAEs) while 4 of 5 subjects in the placebo HD-MAP group reported TEAEs. Overall, 13 TEAEs were reported and all were classified as mild in severity. Further, of these, 5 of 5 TEAEs reported in the active group were classified as probably -study-drug related, while 2 TEAEs in the active HD-MAPs were reported as possibly related to study drug administration. No subject withdrawals were recorded for the 10 subjects.

**Fig 2:**
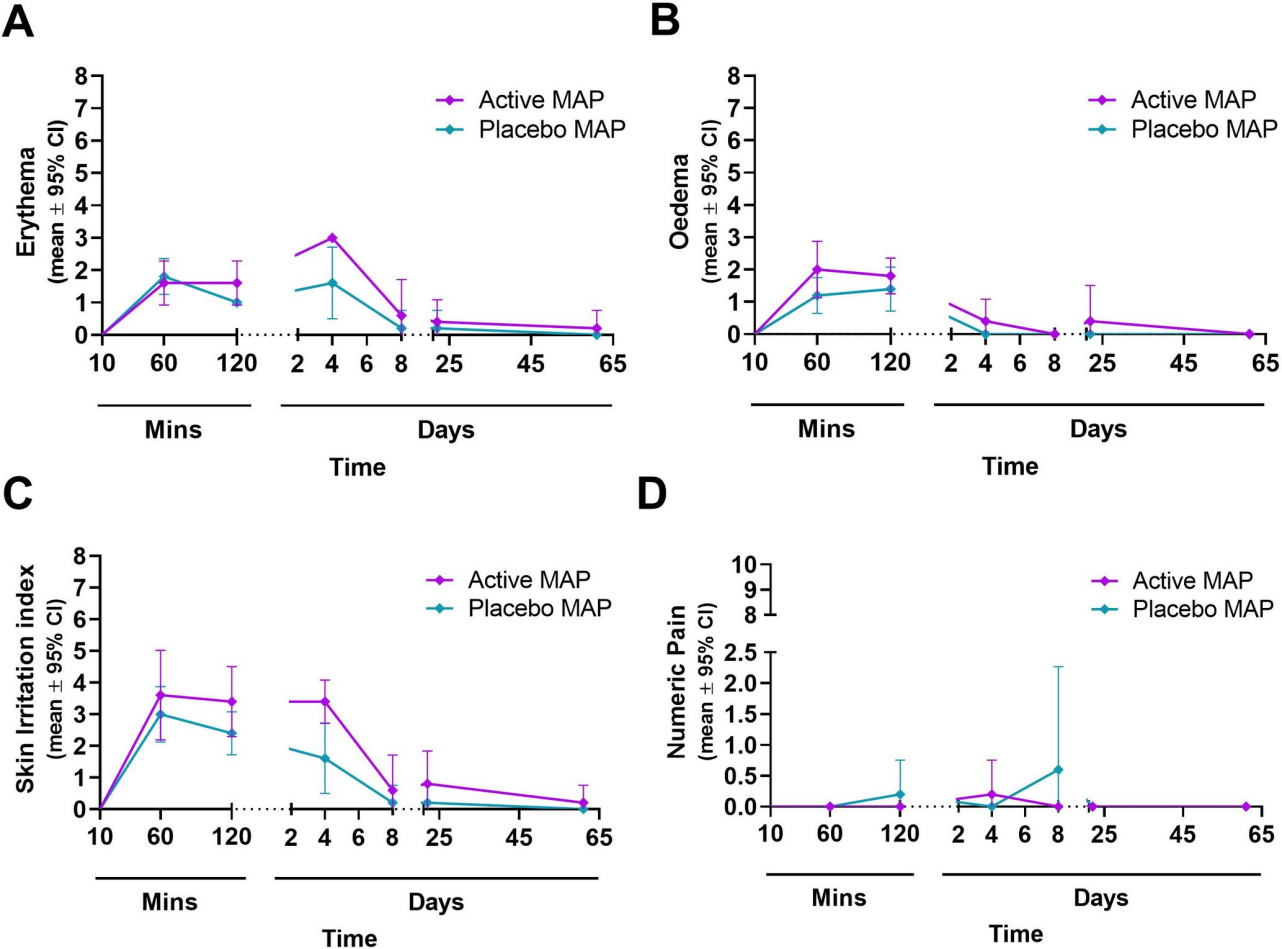
Skin site assessment post HD-MAP applications in the 10 biopsy-group subjects. (A) Erythema scores; (B) Oedema scores; (C) Skin Irritation Index (erythema and oedema scores); (D) Pain scores following HD-MAP applications over the course of the study. Only scores of site 2 were included as it was the only HD-MAP application site that was not biopsied. Depicted are mean ± 95% confidence interval.

**Fig 3:**
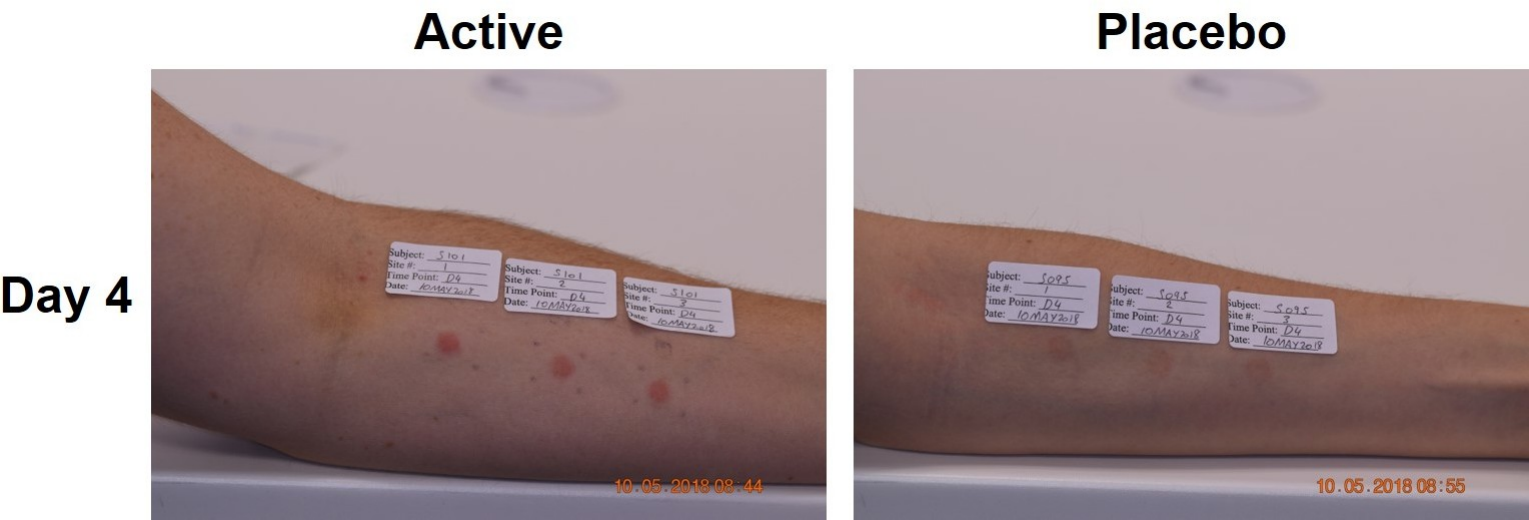
Skin site of two representative subjects post HD-MAP application. Shown are representative volar forearms from active (left) and placebo HD-MAP groups (right). Day 4 images were taken prior to the biopsy.

In addition to assessing AEs, skin reactions (for the non-excised site) were assessed for erythema and oedema. Both were scored (on a scale of 0–4) and combined to generate a Skin Irritation Index (SII) as described previously [12, 17]. There was a short-lived oedema response to HD-MAPs, followed by a delayed erythema (Fig 2A and 2B) peaking by day 4 and returning to baseline by day 8. Combined as SII (Fig 2C), responses were comparable to results reported in earlier clinical trials [12, 17], and as observed in the main part of this trial [19]. The pain scores were minimal on a scale of 0–5 (Fig 2D). Representative images of applicate site reaction on day 4 are shown in Fig 3, whereas a full time-course until resolution is shown in S1 Fig.

### Antibody responses

Immune responses, specifically serum antibodies to influenza, following the application of HD-MAPs were assessed for subjects in the biopsy groups by haemagglutination inhibition (HAI) assay. HAI assessment confirmed that all subjects receiving the active HD-MAP generated clinically relevant immune response while subjects receiving the placebo HD-MAPs did not (Table 2). All subjects in the active HD-MAP group were seropositive at day 22, and 80% seroconverted between day 1 and day 22 (Table 2). The HAI results were consistent with the findings of the main study, except GMT-fold-increases, which were lower in the biopsy groups than the dose-matched groups in the main study [19], most likely due to the small sample size.

**Table 2:** Summary of HAI assay results as geometric mean titres (GMTs), seroprotection (%), seroconversion (%) and GMT-fold increase.

		Haemagglutination inhibition assay					
Treatment group	Assessment	n	day 1	day 4	day 8	day 22	day 61
**Active** (MAP-FA-15)	GMT (± 95% CI)	5	13 (4–49)	13 (4–49)	80 (21–312)	139 (45–428)	106 (49–228)
	Seroprotection (%)		40.0	40.0	**80.0**	**100.0**	**100.0**
	Seroconversion (%)			0.0	**40.0**	**80.0**	**80.0**
	GMT-fold-increase			1.0	**6.1**	**10.6**	**8.0**
**Placebo** (MAP-FA-0)	GMT (± 95% CI)	5	5 (5–5)	5 (5–5)	5 (5–5)	5 (5–5)	5 (5–5)
	Seroprotection (%)		0.0	0.0	0.0	0.0	0.0
	Seroconversion (%)			0.0	0.0	0.0	0.0
	GMT-fold-increase			1.0	1.0	1.0	1.0

95% CI = 95% confidence interval; GMT = geometric mean titre; Seroconversion rate defined as percentage of participants with antibody titre ≥1:40 post-vaccination for initially seronegative (<10) participants, or ≥4 fold the pre-vaccination antibody titre for initially seropositive (≥10) participants; seroprotection rate defined as percentage of participants with antibody titre ≥1:40; Bold typeface indicates CHMP criteria have been met or exceeded (seroconversion rate ≥40%; seroprotection rate ≥ 70%, GMT fold-increase ≥ 2.5). Data where applicable represented as geometric mean ± 95% CI.

### Measurement of skin strata

**Fig 4:**
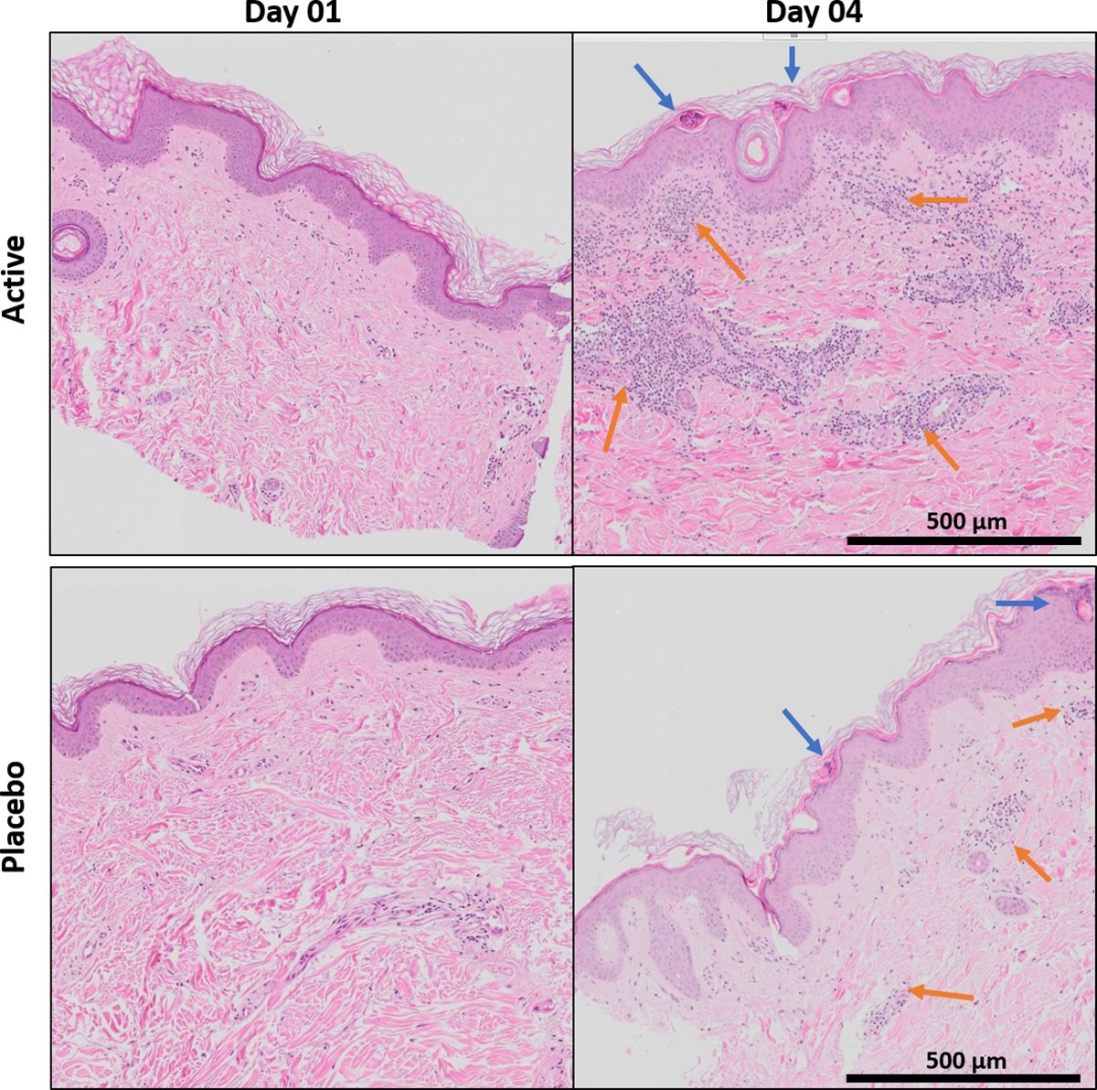
**H&E-stained skin biopsies from two representative subjects who received active/vaccine-coated HD-MAPs (upper row) or placebo HD-MAPs (lower row).** Biopsies were taken aseptically on day 1 (pre) and day 4 (post) HD-MAP application. Following fixation, biopsies were paraffin-embedded, and 5 μm thick sections were subjected to H&E staining. Pre-MAP sections (left column) showed very little cellular infiltration, whereas post-MAP applications (right column) resulted in substantially more cellular influx (dark spots) within the dermis. Epidermal thickening was also noticeable on day 4, along with scab formation in the stratum corneum (SC), and viable epidermis (VE). Blue arrows: micro-scabs post HD-MAP projections penetrating the skin; orange arrows: cell infiltrates. Scale bar: 500 μm.

Following inspection of the H&E-stained biopsies (Fig 4), the thickness of the viable epidermis (VE) was measured. All samples presented with an increase from pre- to post-HD-MAP application (day 1 and day 4, respectively) with no apparent difference between active and placebo groups (S3 Fig). Due to the variability in depth of the dermis achieved by the biopsy and processing, analyses on dermal thickness were not performed.

### Cellular infiltration at the HD-MAP application site

The H&E stained biopsies revealed cellular infiltration in both active and placebo HD-MAP groups following-HD-MAP application on day 4. Representative images from subjects that received active HD-MAPs or placebo HD-MAPs are shown in Fig 4. The *stratum corneum* was present on most samples, intact on day 1, and as expected, partially disrupted post-HD-MAP application (day 4). Micro-scabs, which aligned with the spacing of HD-MAP projections entering the skin (blue arrows), were seen on multiple day 4 biopsy samples. The VE was present in all subjects and timepoints, whereas the dermis, although present in all samples, varied greatly in depth between subjects and individual biopsies. This variation, which was also seen in the pre-HD-MAP application biopsies, was a result of sample collection and processing, and not related to the actual HD-MAP application.

**Fig 5:**
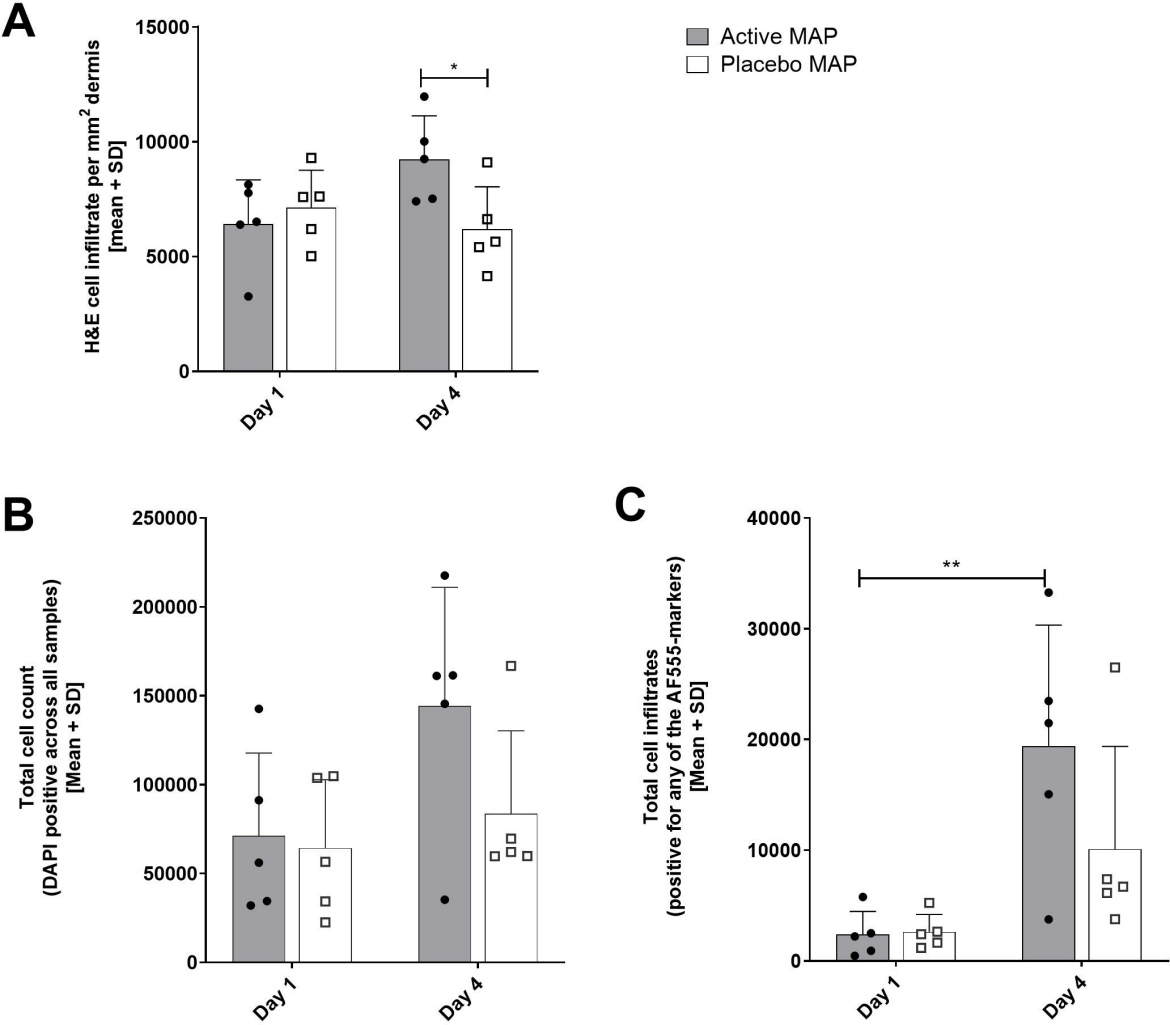
Quantification of cellular infiltration into the skin of active and placebo HD-MAP, pre- and post-HD-MAP application. (A) Haematoxylin-stained nuclei as proxy for cell infiltrates were indirectly quantified by dividing total cell area measurements by average cell size, and then normalised to the dermal area. (B) Total (DAPI stained) cells per group and time-point. (C) Cumulative cell counts of AF-555-positive stained cells per group and timepoint, where cells positive for specific markers were added up per group and time point. A marked increase in cellular infiltrate in active HD-MAP-treated subjects at day 4, compared to day 1, and compared to the placebo group on both days. Statistical significance was determined by two-way ANOVA; * p<0.05, ** p<0.01.

Cell infiltration was characterised by small, dark haematoxylin-stained cell nuclei, predominantly found within the dermal layers of the skin (Fig 4). As expected, more infiltrating cells were found post application of active HD-MAPs by day 4 in the dermis when compared against placebo HD-MAPs (Figs 4, yellow arrows, and 5A). No significant difference in total infiltrating cells were seen between day 1 and day 4 in the placebo HD-MAP group subjects (Fig 5A). In summary, micro-scab formation post-HD-MAP application was seen in both treatment groups, whereas cellular infiltration was more pronounced in the active HD-MAP group on day 4.

For IF, additional sections were counterstained with DAPI and eleven AF-555 conjugated markers (S1 Methods) quantified for total and specific cell infiltration (S4 Fig). In agreement with the H&E cell infiltration data, an increase in total cell counts was seen at day 4 post active HD-MAP application in four of five subjects, but not the placebo HD-MAP group (Fig 5B). Next, the total number of AF-555-positive cells (termed ‘infiltrates’) was determined to quantify the total cellular infiltrate (Fig 5C). There was a statistically significant increase (p<0.01) in total cell infiltrates at day 4 in the active HD-MAP group when compared to day 1 (pre-HD-MAP application). The placebo HD-MAP group showed a slight increase of cell infiltrates at day 4. Representative images of all IF markers for two subjects are provided in S4 Fig. In summary, an increased number of cells infiltrated tissue post active HD-MAP application when compared with placebo HD-MAPs.

### Quantitation of immune cells determined by flow cytometry

**Fig 6:**
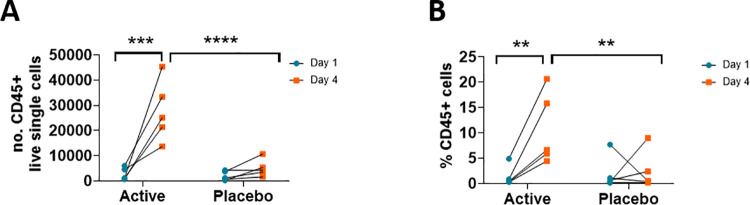
Increase of haematopoietic cells into the application site of antigen-coated HD-MAPs between day 1 and day 4. Haematopoietic cells measured by the presence of CD45 cell surface marker in human skin biopsies taken pre- (day 1 blue) and 3 days (day 4 orange) post HD-MAP application as (A) total cell counts or as (B) percentage of CD45+ cells of all single live cells. Analysed by two-way ANOVA, * p<0.05, *** p<0.001, **** p<0.0001.

To complement histological assessment of skin biopsies, flow cytometric analyses using 14 cell surface markers were performed, with the gating approach shown in S2 Fig. Firstly, the skin biopsy samples were assessed for the presence of haematopoietic cells (CD45^+^, Fig 6). There was a significant increase in total number and % of CD45^+^ cells (Fig 6A and 6B) present in the biopsy samples taken from the active HD-MAP group at day 4 compared to placebo HD-MAP group (*p*<0.05). There was a significant increase in the number and frequency of CD45^+^ cells from day 1 to day 4 in the active HD-MAP group, but not in the placebo group, indicating an influx of haematopoietic cells into the application site of antigen-coated HD-MAPs by day 4.

**Fig 7:**
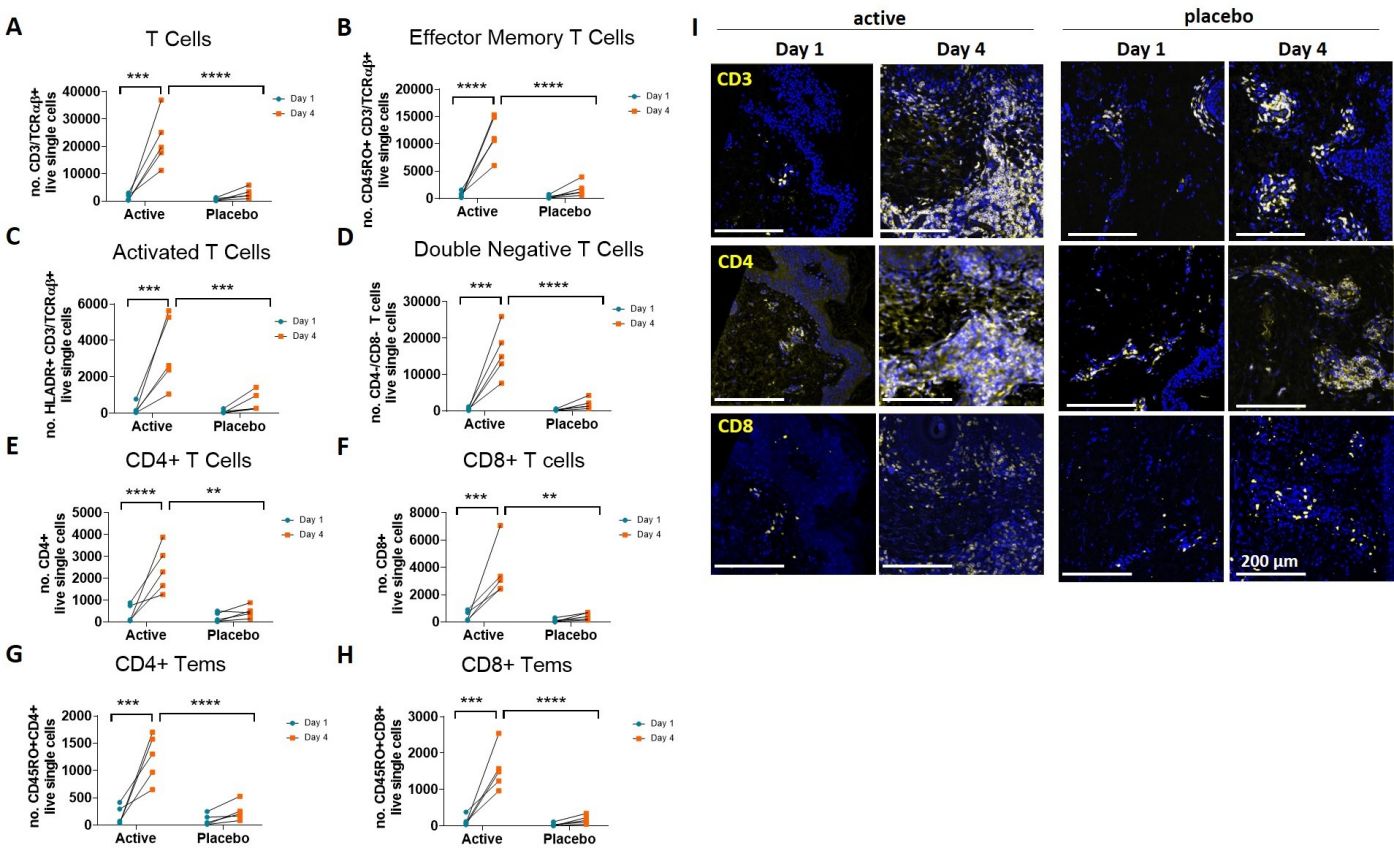
Influx of T cells into the application site of antigen-coated HD-MAPs on/by day 4. (A) CD45+ population sub-gated for CD3/TCRαβ+ T cell populations in human skin biopsies taken pre- (day 1 blue) and 3 days post- (day 4 orange) HD-MAP application. (B) T cells were further assessed by sub-gating for the presence of CD45RO to quantify effector memory T cells (Tems) in peripheral tissue; (C) HLA-DR, a late activation marker for T cells; (D-H) CD4 and CD8 T cell subtype cell surface markers. (I) Immunofluorescence slides are from two representative subjects who received active HD-MAP (left) or placebo HD-MAP (right) from day 1 and day 4. Biopsies were taken aseptically on day 1 and 4 HD-MAP application. Following fixation, paraffin-embedded 5 μm thick sections were subjected to immunofluorescent staining. DAPI (blue), specific cell surface markers (yellow). Tem: effector memory T cells. Statistical significance assessed by two-way ANOVA, * p<0.05, ** p<0.01, *** p<0.001, **** p<0.0001.

Next, CD45^+^ cells were sub-gated into T cells based on the presence of both CD3 and TCRαβ, and further separated into CD4^+^ and CD8^+^ T cell populations. Based on the selected cell surface markers, T cells were classified as effector memory T cells (Tem) by CD45RO expression and for activation levels by HLA-DR expression [21]. CD4^+^ and CD8^+^ T cell subsets were also further sub-gated to assess the presence of tissue resident memory T cells (Trm) distinguished in skin by the presence of the early activation marker CD69 plus αEβ7 integrin (CD103) [22]. In line with the overall increase in CD45^+^ cells, a significant increase in CD3^+^/TCRαβ^+^ T cells present in the skin biopsy digest was seen on day 4 in the active HD-MAP group compared with the placebo group (Fig 7A and 7I). Likewise, CD4^+^ and CD8^+^ T cells were also significantly increased (Fig 7E and 7F, respectively, and Fig 7I), as was the frequency of activated T cells (Fig 7C), which correlated well with the IF images (Fig 7I). Across the T cell populations assessed, Tems made up 50% of all T cells including the CD4^+^ and CD8^+^ subtypes pre- and post HD-MAP application (both active and placebo; Fig 7G and 7H, respectively), and the frequency of these cells increased in the active but not placebo group. However, comparison of active vs placebo data was not performed for Trms as these cells were not detected in multiple pre-application samples. There was also an increase in CD3^+^ CD4^-^ CD8^-^ (double negative T cells), particularly in subjects in the active group. This could reflect an increase in CD3^+^ gamma-delta T cells. Overall, the results indicated that there was a significant influx of both CD4^+^ and CD8^+^ T cells into the application site of active HD-MAPs at day 4 compared to day 1, but not in the application sites of placebo HD-MAPs.

**Fig 8:**
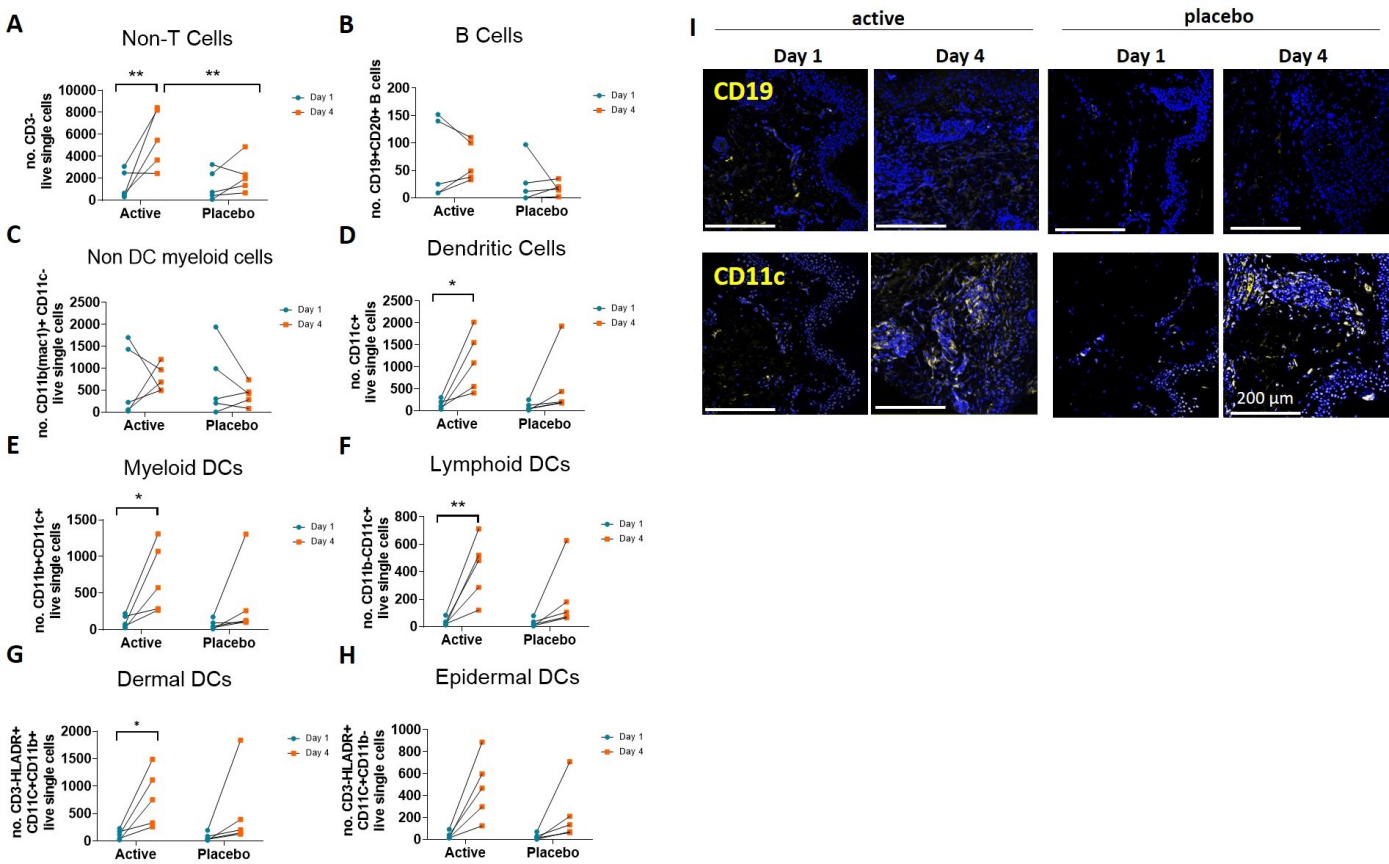
Influx of non-T cells and DCs into the application site of antigen-coated HD-MAPs by day 4. (A) CD45^+^ population sub-gated for CD3/TCRαβ- non-T cell populations in human skin biopsies taken pre- (day 1 blue) and 3 days (day 4 orange) post HD-MAP application. (B) Non-T cells were further sub-gated into B cells (CD19^+^/CD20^+^). (C) Non-DC myeloid cells (CD11b^+^CD11b^-^). (D) DCs (CD11c^+^) and corresponding subtypes of (E) myeloid (CD11b^+^) and (F) lymphoid (CD11b^-^) populations. The presence of HLA-DR was used to distinguish (G) dermal CD11c^+^CD11b^+^ and (H) epidermal CD11c^+^CD11b^-^ DCs. Statistical significance was assessed by two-way ANOVA, * p<0.05, ** p<0.01.

There was also an increase of CD45^+^ non-T cells (CD3^-^/TCRαβ^-^) present in the skin biopsy digest (Fig 8A) on day 4 in the active HD-MAP group compared with the placebo group. Statistical significance between treatment groups at day 4 was only observed in the total number, not % of live cells for CD3^-^ non-T cells present in biopsies. While B cells (CD19^+^/ CD20^+^, HLA DR^+/-^) were quantified, counts were low and did not differ significantly between treatments and/or time-points (Fig 8B and 8I). Non-T cells (CD3-/TCRαβ-) were further separated into non-DC myeloid cells (CD11b^+^ CD11c^-^, Fig 8C) and CD11c^+^ DCs (Fig 8D); the latter further subgated based on CD11b^+/-^ expression into myeloid (CD11c^+^ CD11b^+^) and lymphoid (CD11c^+^ CD11b^-^) DCs (Fig 8E and 8F). Dermal and epidermal DCs were subtyped based on the expression of HLA-DR (Fig 8G and 8H). The overall number of DCs increased on day 4 in the active group (Fig 8I). This increase was seen in all DC subgroups but was only statistically significant for CD11c^+^ (myeloid and lymphoid) DCs. There was no significant difference between active and placebo groups on day 4. In addition, no significant differences between treatment or time in other myeloid cell types (CD11b^+^ CD11c^-^; macrophages, granulocytes, and NK) were seen in the skin biopsies (Fig 8C). An overview of the changes in marker expression by flow cytometry is summarised in Table 3. Taken together, these results indicate that myeloid and lymphoid DCs increased by day 4 in the active group, whereas no significant infiltration into or migration from the application sites by non-dendritic myeloid cells was observed.

**Table 3:** Summary of changes in marker expression between pre (day 1) and post (day 4) MAP application.

Change from day 1 to day 4	Change from baseline	
	Active	Placebo
**Total haematopoietic cells**		
Haematopoietic cells (CD45^+^)	↑↑↑	-
**T cell subsets**		
CD4+ T cells (CD3^+^. CD4^+^)	↑↑↑	↑
CD8+ T cells (CD3^+^, CD8^+^)	↑↑↑	↑
TEM (CD3^+^, CD45RO^+^)	↑↑↑	↑
TRM (CD3^+^, CD69^+^, CD103^+^)	*	*
Activated T cells (CD3^+^, HLA DR^+^)	↑↑↑	↑
**B cells**		
B cells (CD19^+^/CD20^+^)	ND	ND
**Dendritic cells**		
Myeloid DC (CD45^+^, CD11b^+^, CD11c^+^)	↑↑↑	↑
Lymphoid DC (CD45^+^, CD11b^-^, CD11c^+^)	↑↑↑	↑
**Other myeloid cell types**		
**Other myeloid cell types** (CD45^+^, CD11b^+^, CD11c^-^)	-	-

Skin biopsies were subjected to flow cytometric analysis of single cell suspensions. Arrows represent minor (↑) or extensive (↑↑↑) increase;—represents no change; ND, not detected* no comparison performed due to lack of day 1 data.

### Distribution of immune cells by immunofluorescent histology

**Fig 9:**
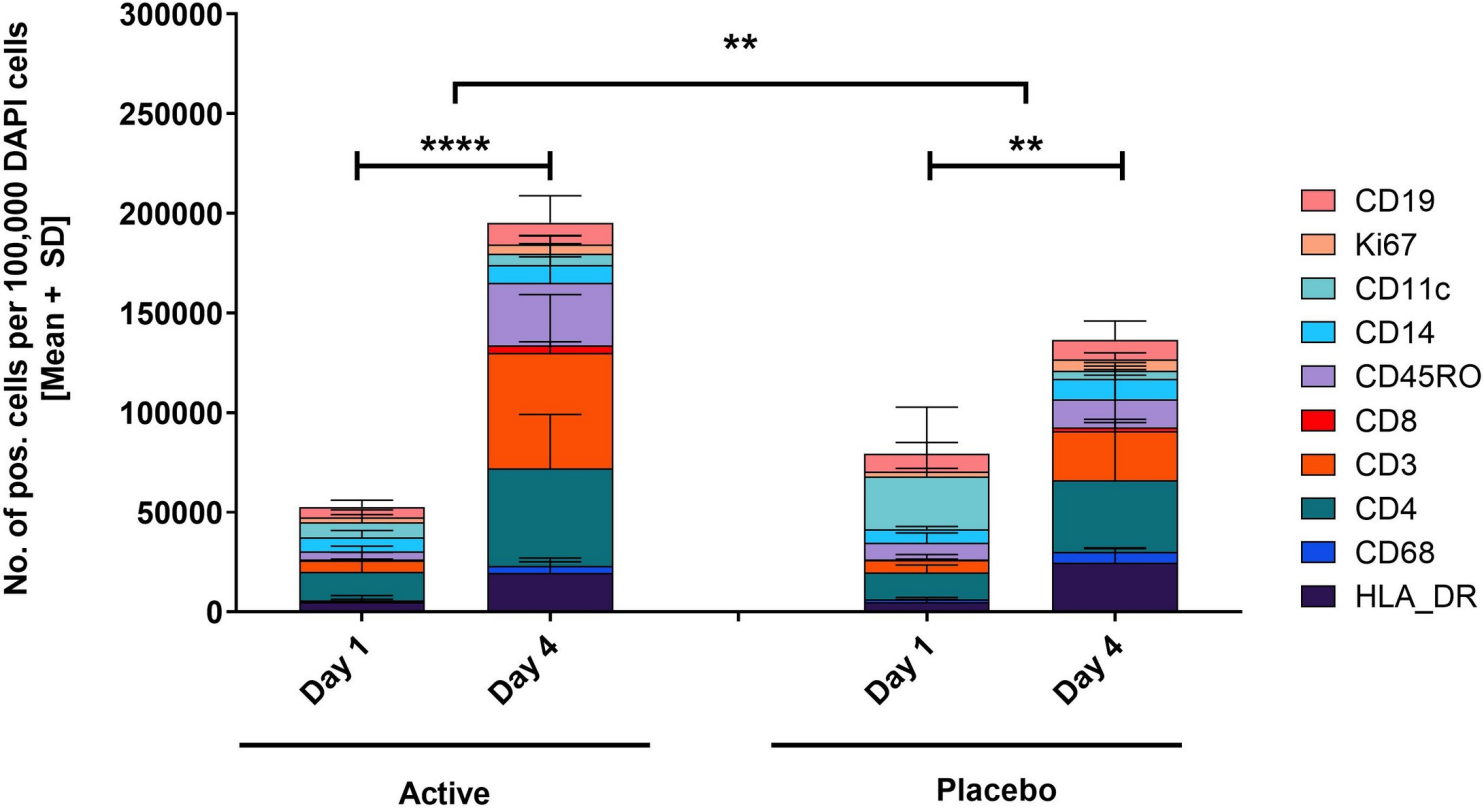
Assessment of cell infiltrates. Cells that stained AF555-positive for CD3, CD4, CD8, CD11c, CD14, CD19, CD45RO, CD68, HLA-DR, and Ki-67 were quantified and normalised against DAPI stained cells. Statistical significance was assessed by two-way ANOVA matched by subject on log transformed data; placebo day 1 to day 4 ** p = 0.0011, active day 1 to day 4 **** p<0.0001, active versus placebo day 1 to day 4: ** p = 0.0084.

Tissue matrix arrays (TMAs) were analysed by single, not multiplex IF staining. Representative images for all markers and one subject per treatment group are shown in S4 Fig. IF assisted primarily examining the site of cellular influx following HD-MAP application (S4 Fig) and separately enumerating cells (Fig 9). Overall, a significant higher level of cellular influx was seen post active HD-MAP application by day 4 relative to day 1 than in the placebo HD-MAP group (*p* = 0.0084; Fig 9), consistent with what was seen by flow cytometry. Comparisons of cellular influx in active versus placebo samples from day 1 to day 4 were also significantly different (*p* = 0.0084). Overall, in line with the flow cytometric data, it was evident that there was a higher level of cellular influx seen post active HD-MAP application by day 4 than in the placebo HD-MAP group.

HLA-DR, a marker expressed mostly by B cells, macrophages, APCs but also activated T cells, was expressed throughout the dermis, with an emphasis on the papillary dermis on day 4 (S4 Fig). In line with the flow cytometric analysis of CD3^+^ T cells (Fig 7A and S4 Fig), the IF staining clearly demonstrated an increase in CD3^+^T cells in the dermis by day 4 in the active HD-MAP group when compared to the placebo group. Similarly, CD4^+^ and CD8^+^ were found markedly upregulated in the dermis of subjects post-HD-MAP application. Interestingly, CD45RO^+^ cells were predominantly located in the papillary dermis, with some clustering apparent in the reticular dermis (S4 Fig). The number of cells expressing the monocyte and macrophage marker CD68 was also elevated following HD-MAP application throughout the dermis, but no clear distinctions between active and placebo groups were noticeable (S4 Fig). Likewise, CD14^+^ (monocytes and macrophages) and CD11c^+^ (monocytes, granulocytes, a subset of B cells, DCs and macrophages) cells infiltrated both active and placebo HD-MAP application sites dispersed throughout the dermis (S4 Fig), with some clustering in the vicinity of hair follicles. By contrast, the proliferation marker Ki-67 was upregulated in the post-HD-MAP application groups predominantly present in the basal layer of the VE, with some scattered cells in the dermis and around hair follicles (S4 Fig). Cells expressing B cell markers CD19 and CD20 were too low for accurate quantification, irrespective of HD-MAP treatment group or time point. A summary of changes in marker expression are summarised in Fig 9. Taken together, HD-MAP application resulted within the dermis in HLA-DR^+^, CD3^+^, CD4^+^, CD8^+^, CD45RO^+^, CD11c^+^ and CD14^+^ cell influx, as well as upregulation of Ki-67 by day 4 in the VE; antigen-coated HD-MAPs appeared to further increase the presence of HLA-DR^+^, CD3^+^, CD4^+^, CD8^+^, CD45RO^+^ cells by day 4 post application (S4 Fig).

## Discussion

This study is, to the best of our knowledge, the first clinical study published assessing local application-site responses at the cellular level following HD-MAP applications to the skin. To date, studies of this type have been conducted only in animal models, which suggested increased local inflammation, increased local cytokine/chemokine production, and antigen uptake and migration of matured DCs away from the skin, amongst others [23–25].

At the macroscopic level, there were noticeable different effects on the skin between the application sites of placebo and active HD MAPs. This was expected based on local skin responses reported by others following microneedle/MAP applications [12, 14]. Since stronger and longer lasting skin responses were seen in the active group, this suggests that a considerable part of the responses was related to the vaccine rather than the HD-MAP projections alone. Likewise, the data from the flow cytometry and IF showed significant increase of T cells in the biopsies from the active but not the placebo HD-MAP groups. Given the 3-day timeframe between application and biopsy, this indicated an induction of an adaptive T cell memory response rather than a primary response.

Challenges to the interpretation of our results were created by the use of influenza vaccine in this study, since all patients were likely to have been pre-exposed to influenza, even if the pre-vaccination HAI titres were < 1:40. The T cell infiltration was in line with findings presented by Forster *et al*., where an increase in the frequency of circulating influenza-specific CD4^+^ T cells capable of secreting IFN-γ, IL-2 or TNF-α was detected [19]. However, no correlation could be made between the magnitude of skin response and the T cell or antibody responses either at baseline or post-vaccination. Pre-clinical studies have demonstrated significant CD8^+^ T cell responses to ovalbumin [26], or Tem and CD8^+^ T cell responses to live, virus or viral vector vaccines [27, 28] when delivered by MAPs. Yet, the influx of CD8^+^ cells was surprising, given that Chua *et al* showed that split influenza vaccines did not induce CD8 responses [29]. We could not distinguish between general T cell and influenza antigen-specific T cell influx, so the increase in the T cell population could have been the result of bystander-recruitment, although we did not see increases in other populations such as B cells and non-dendritic cell myeloid cells. It would be interesting to look at the CD4/CD8 T-cell profile in skin at the application site of HD-MAP vaccinations containing live-attenuated virus vaccines, for example, measles, mumps, rubella, that can replicate in the host cells.

In pre-clinical studies, microneedles and MAP technologies have shown that microinjuries inflicted by the projections penetrating the skin can have immunostimulatory effects [16, 30]. The skin’s cellular responses observed in this study were consistent with a previously established hypothesis of cell death and PAMPs together enhancing immunogenicity [16, 31]. Based on current knowledge, cutaneous vaccine deliveries by MAP and NP have been shown to lead to increased cell infiltration into the tissue [24, 27, 32]. This cellular infiltration, controlled inflammation and cell death play a pivotal role in stimulating DCs and enhancing transcutaneous immunisations [16, 33]. However, we did not observe a direct correlation between the degree of the local skin response (erythema, cell influx) and measured antibody responses [19].

Whilst MAPs will cause microinjuries upon insertion into the skin, varying projection densities and different application procedures are likely to generate different cellular response patterns in nature and degree manifested by different levels of erythema or oedema [12]. This level of variation will most likely be further influenced by the type of excipient and the antigen delivered. For example, stronger and longer-lasting skin responses were seen post epidermal delivery of gold particle-mediated [34] and alum-adjuvanted Hep B administered ID [35]. Yet, based on the patient compliance reported by Rouphael *et al*., MAPs in general are well tolerated and accepted [14].

Several limitations of the study must be acknowledged. Firstly, the subjects were likely to have been pre-exposed to influenza, either by infection and/or previous vaccination. Therefore, the cellular responses observed in this study might reflect the response to HD-MAPs delivering antigens that a recipient was primed. It is also possible that the cellular infiltrate observed was due to a non-influenza component in the vaccine, such as egg-protein, that was not present on the placebo HD-MAP. With only 5 subjects per group, the interpretation of results is limited and hypothesis generating in nature. Although significant differences were observed, the sample size wasn’t powered to draw definitive conclusions. Finally, cellular migration and activation were assessed at only one time point after vaccination. Therefore, we have only a limited understanding of what is happening on the cellular level in the skin post HD-MAP application and/or immunisation. It would be informative to investigate skin application sites at a range of earlier and later time points.

Pre-clinical studies suggested a physical immune-enhancing effect of the HD-MAP [16], and clinical studies would benefit from additional exploratory studies to assess whether the HD-MAP induces responses that resemble the effect of some chemical adjuvants’ but do so by physical interactive mechanisms leading to adjuvantation. Further, the type of vaccine coated on MAPs may stimulate different immune pathways and therefore, it would be useful to investigate immune profiles of MAP vaccination using different types of vaccine (protein antigen, live-attenuated virus, inactivated virions, VLPs, etc), as well as antigens to which the recipient has not had previous exposure.

Based on other vaccine studies, and animal models, the coating formulation deposited within the skin will diffuse as well as be actively taken up by APCs, transported and processed in the draining lymph nodes close to the application site. The excretion mechanism of the antigen(s) has not been studied though and will form part of future investigative studies of pre-clinical origin. We stipulate that the excretion will be antigen-specific but comparable to the e.g. intramuscular injection comparator.

In conclusion, HD-MAP applications resulted in mild visible skin responses at the application site, that included transient erythema and oedema. These were more pronounced when antigen was delivered by the HD-MAP. Application of influenza vaccine-coated, but not uncoated HD-MAPs was associated with an increase of CD3^+^, CD4^+^ and CD8^+^ T cells as well as CD11c^+^ DCs in the skin. The influx of T cells suggests a recall response to the antigen consistent with the fact that the subjects in the study would have had prior exposure to influenza antigens.

## Supporting information

S1 Fig Photographs of HD-MAP application to subjects. (PDF)

S2 Fig Gating strategy. (PDF)

S3 Fig Assessment of skin strata thickness. (PDF)

S4 Fig Immunofluorescence slides from two representative. Subjects who received active/vaccine-coated HD-MAP or placebo HD-MAP.
(PDF)

S1 Table Antibodies used for flow-cytometry analysis of biopsies. (PDF)

S2 Table Antibodies used for immunohistochemistry. (PDF)

S1 File (PDF)

S1 Methods (PDF)
