# Supplementary material for: Cellular responses at the application site of a high-density microarray patch delivering an influenza vaccine in a randomized, controlled phase I clinical trial
Source: PLoS One. 2021 Jul 30;16(7):e0255282. doi: 10.1371/journal.pone.0255282 (PMC8323919; doi:10.1371/journal.pone.0255282)
Supplement: S1 Fig — (PDF) [file pone.0255282.s001.pdf]

S1 Fig. Photographs of HD-MAP application to subjects.

	Active	Placebo
**Pre-application**	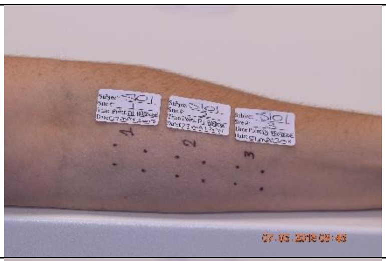	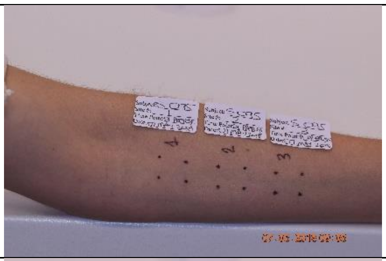
**10 min**	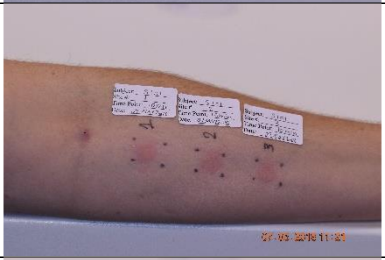	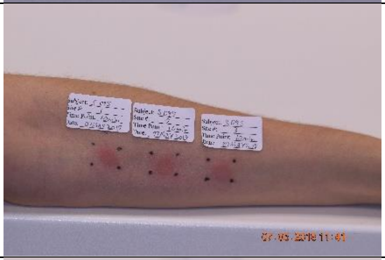
**1 hr**	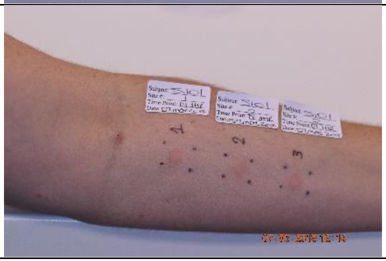	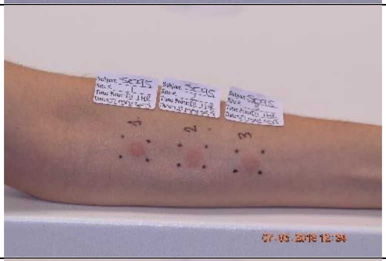
**2 hr**	N/A	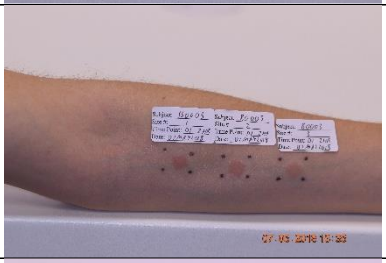
**Day 2**	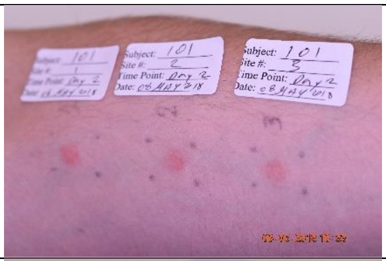	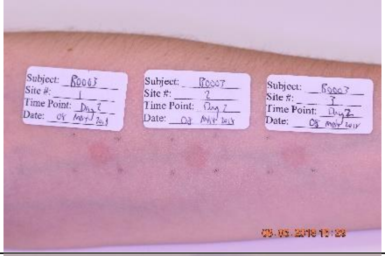
**Day 4**	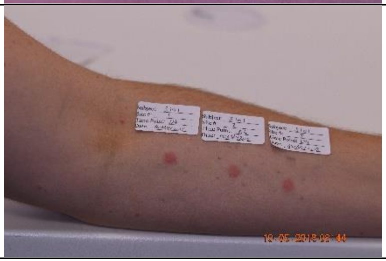	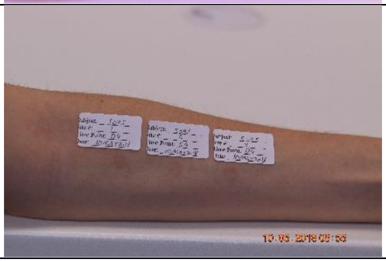

**Day 8**	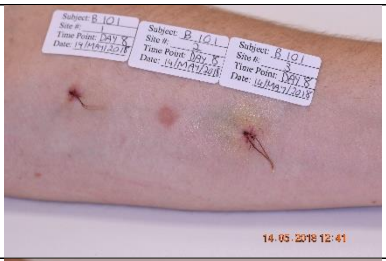	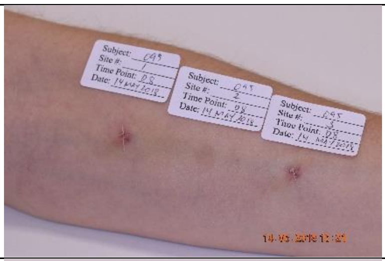
**Day 22**	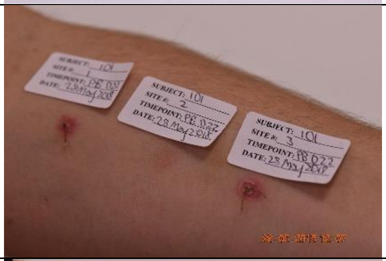	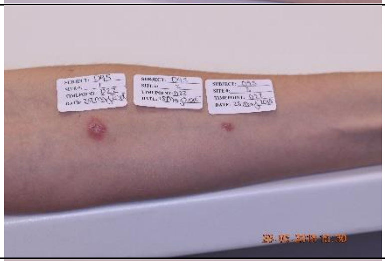
**Day 61**	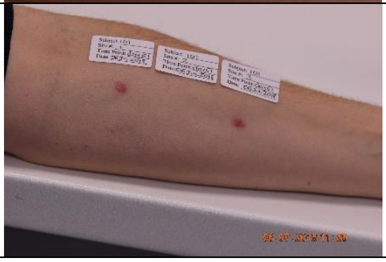	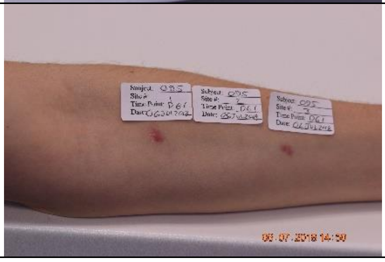

S1 Fig. Photographs of HD-MAP application to subjects. Shown are volar forearm of a representative subject from the active (left column) and placebo HD-MAP application (right column). The day 4 images were taken prior to the biopsy. Following stitching, the biopsy sites were clearly visible on day 22, with surgical dissolving suture material still in place in some subjects. N/A, time-point not available of this subject.
