# Supplementary material for: Cellular responses at the application site of a high-density microarray patch delivering an influenza vaccine in a randomized, controlled phase I clinical trial
Source: PLoS One. 2021 Jul 30;16(7):e0255282. doi: 10.1371/journal.pone.0255282 (PMC8323919; doi:10.1371/journal.pone.0255282)
Supplement: S2 Fig — (PDF) [file pone.0255282.s002.pdf]

S2 Fig. Gating strategy.

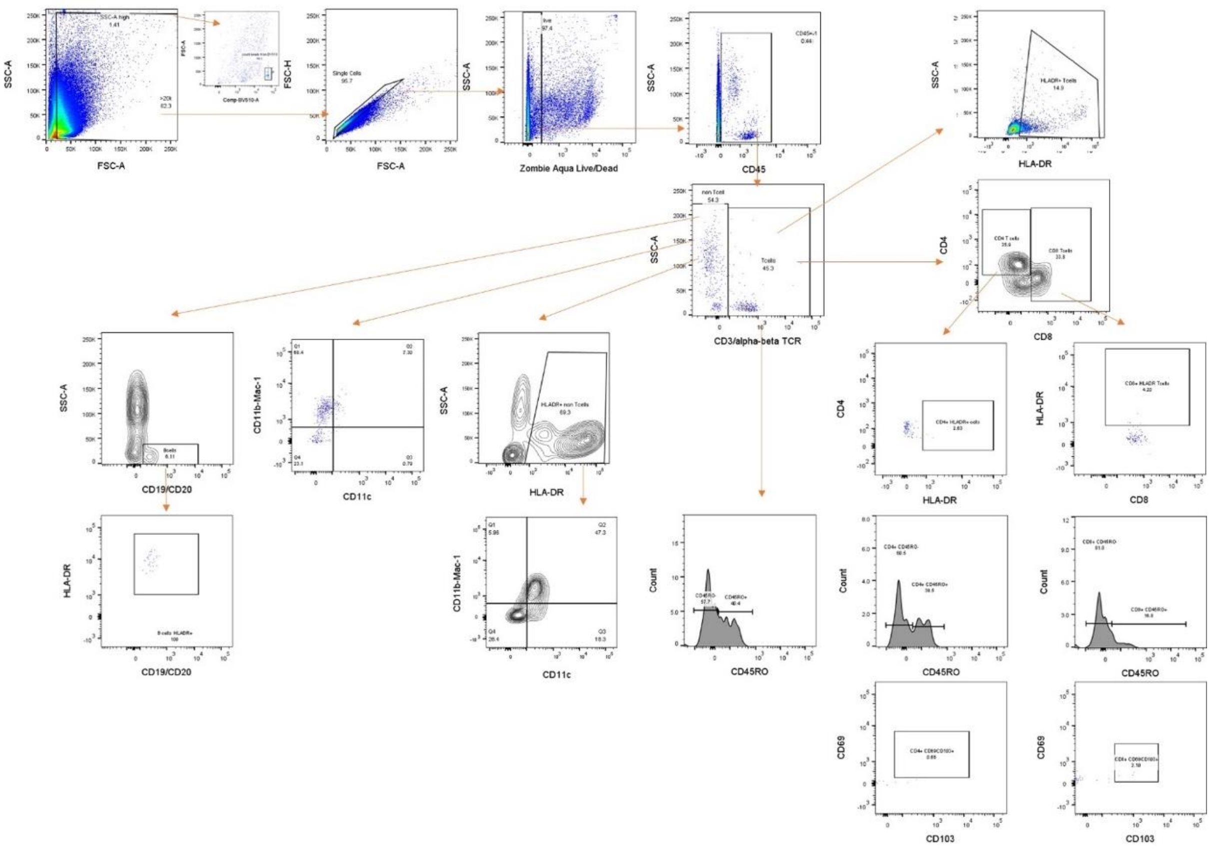

S2 Fig. Gating strategy. In place of a lymphocyte gate, a >20,000 event cut-off gate was used to exclude cellular debris, as back-gating showed that staining for markers of interest fell outside this gate (e.g. monocytes, granulocytes, neutrophils). Next doublet discrimination was performed, followed by gating on live cells. Next, CD3 gates were set of the CD45+ population, and subdivided into CD4+ and CD8+ T cells. Subpopulations of T cells were then assessed for CD45RO and CD103/CD69 expression. The CD3- population was assessed for CD19+/20+ B cells. Separately, HLA-DR, CD11b and CD11c were analysed of the CD45+ CD3- populations.
