# Supplementary material for: Cellular responses at the application site of a high-density microarray patch delivering an influenza vaccine in a randomized, controlled phase I clinical trial
Source: PLoS One. 2021 Jul 30;16(7):e0255282. doi: 10.1371/journal.pone.0255282 (PMC8323919; doi:10.1371/journal.pone.0255282)
Supplement: S3 Fig — (PDF) [file pone.0255282.s003.pdf]

S3 Fig. Assessment of skin strata thickness

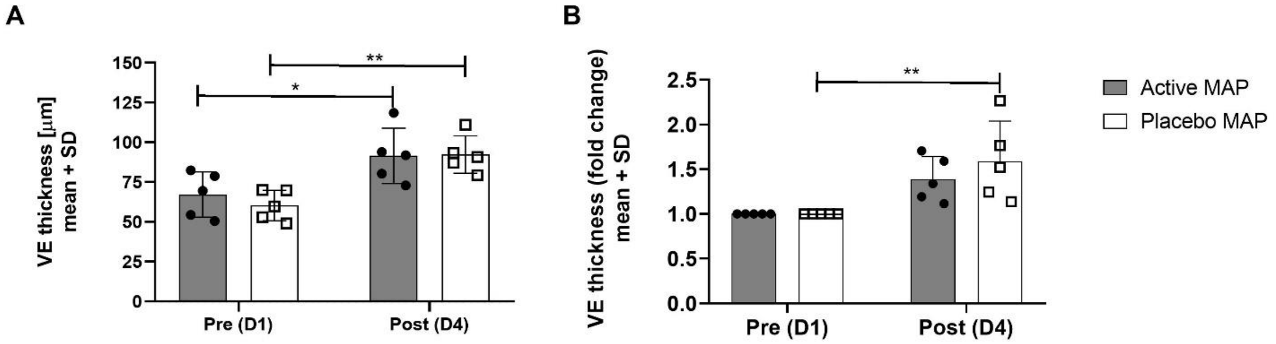

S3 Fig. Assessment of skin strata thickness. Thickness of the viable epidermis (VE) in the active and placebo HD-MAP groups at day 1 and day 4 are shown as total values (A) and fold change (B). Statistical significance was assessed by Two-way ANOVA with Sidak’s multiple comparison; * $p<0.05$, ** $p<0.01$.
