# Supplementary material for: Cellular responses at the application site of a high-density microarray patch delivering an influenza vaccine in a randomized, controlled phase I clinical trial
Source: PLoS One. 2021 Jul 30;16(7):e0255282. doi: 10.1371/journal.pone.0255282 (PMC8323919; doi:10.1371/journal.pone.0255282)
Supplement: S4 Fig — Subjects who received active/vaccine-coated HD-MAP or placebo HD-MAP. (PDF) [file pone.0255282.s004.pdf]

S4 Fig. Immunofluorescence slides from two representative subjects who received active/vaccine-coated HD-MAP or placebo HD-MAP.

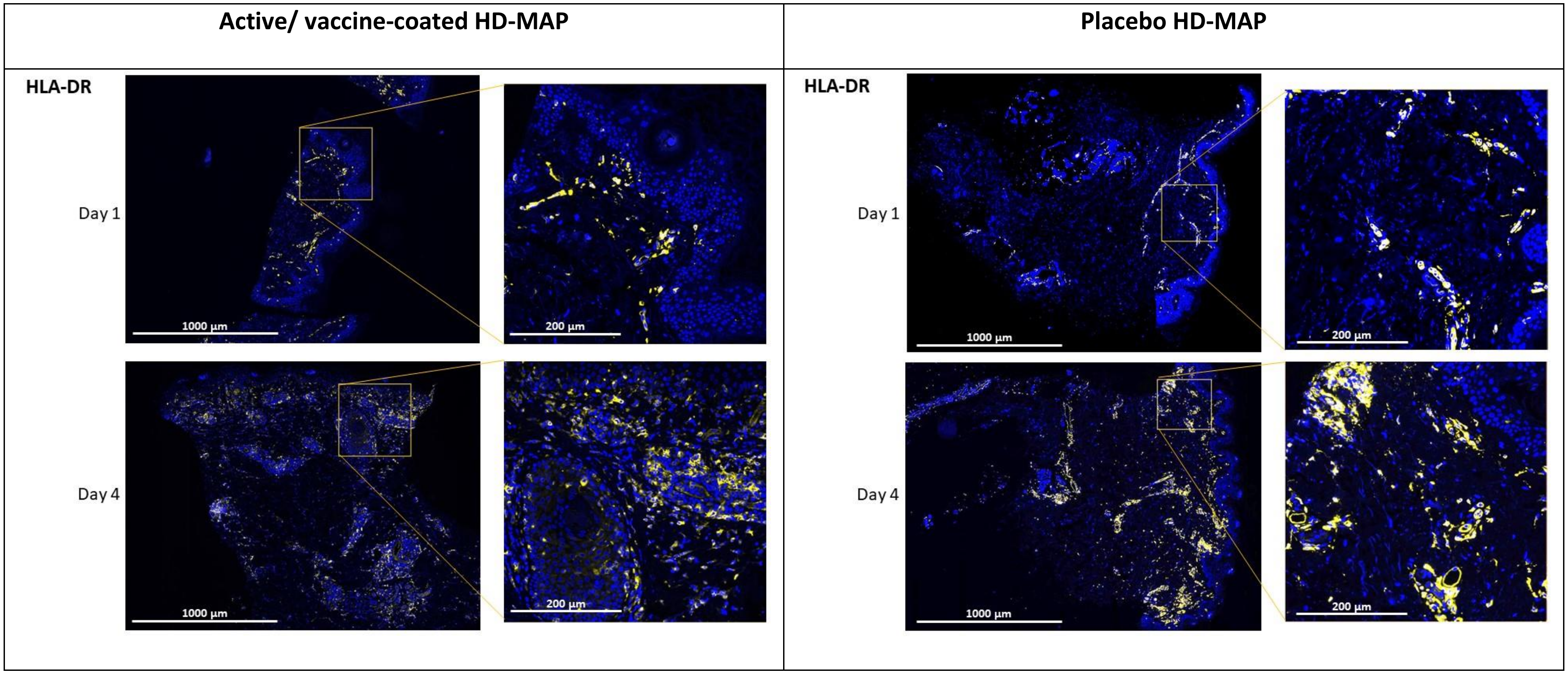

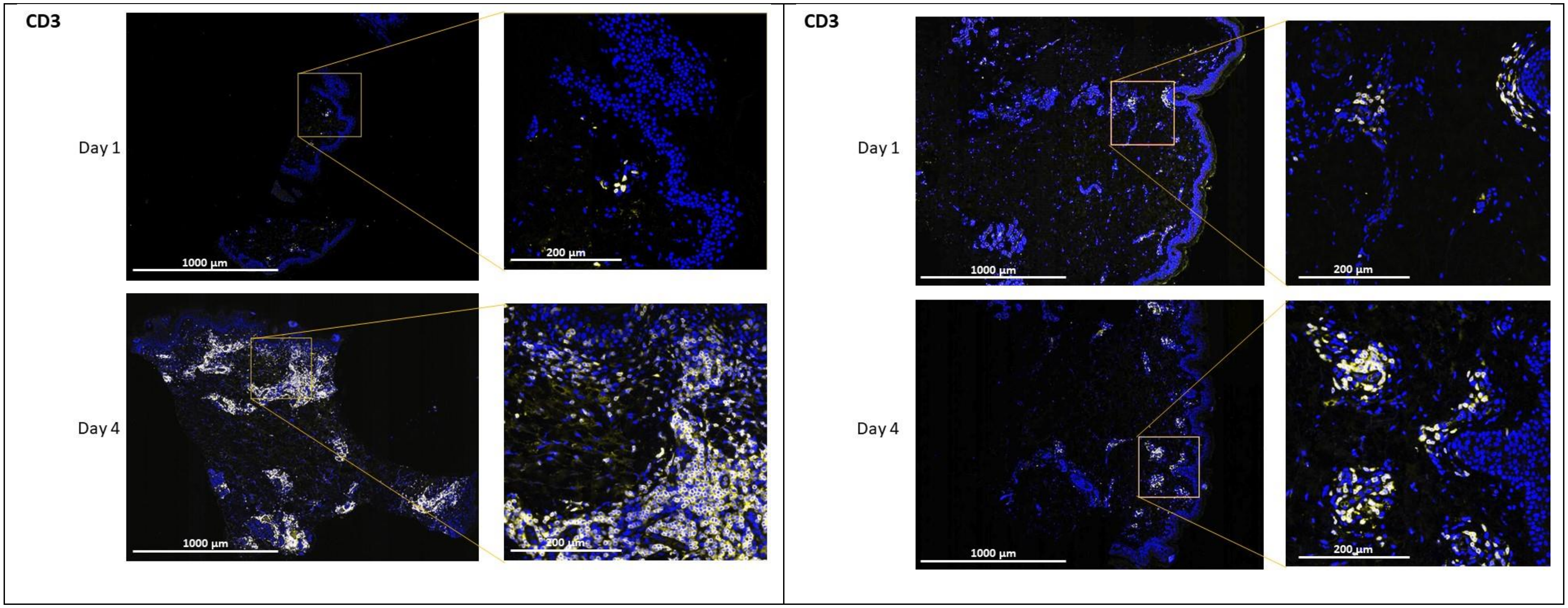
CD3
Day 1
1000 µm
200 µm
Day 4
1000 µm
200 µm
CD3
Day 1
1000 µm
200 µm
Day 4
1000 µm
200 µm

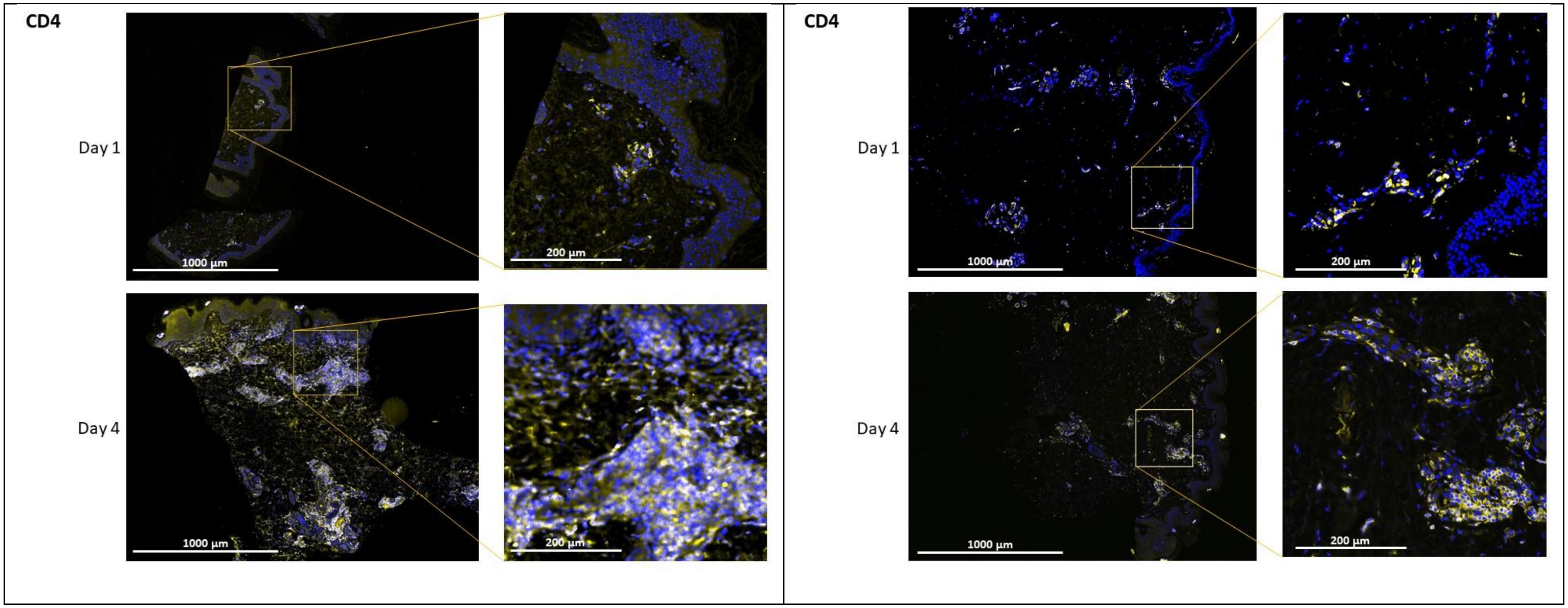
CD4
Day 1
Day 4
1000 µm
200 µm
CD4
Day 1
Day 4
1000 µm
200 µm

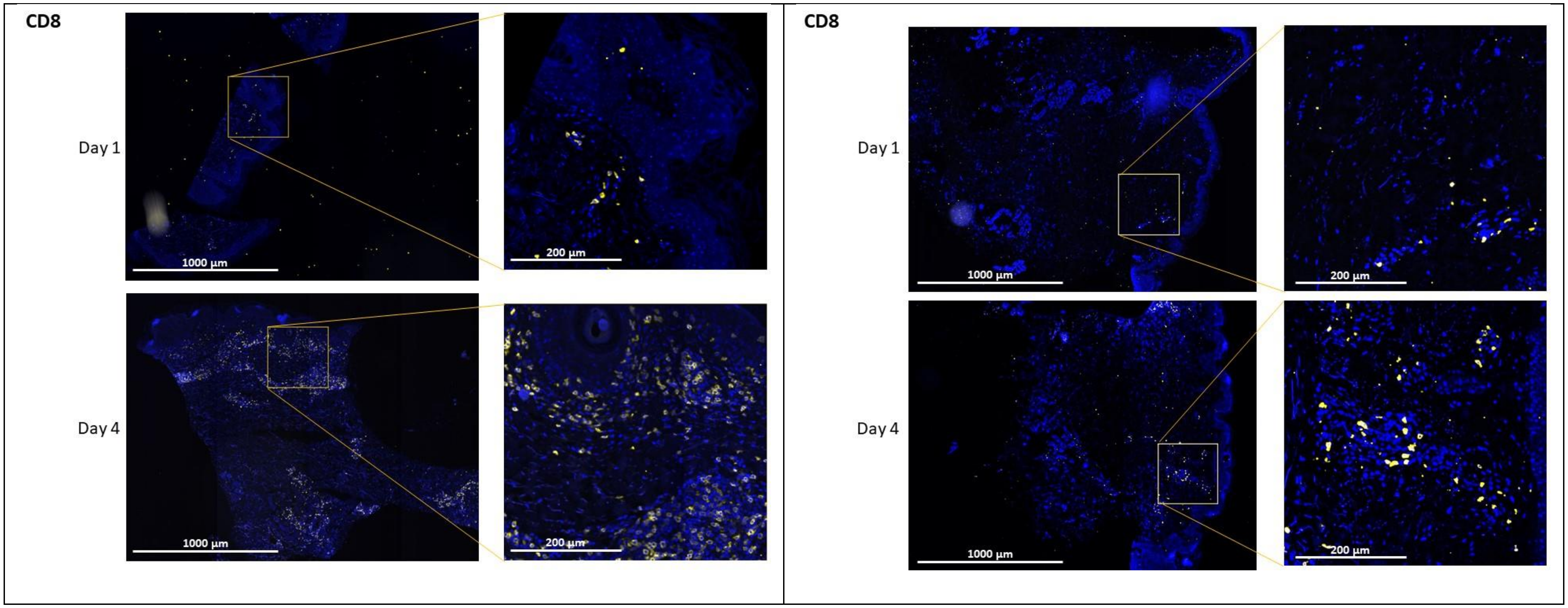
CD8
Day 1
1000 µm
200 µm
Day 4
1000 µm
200 µm
CD8
Day 1
1000 µm
200 µm
Day 4
1000 µm
200 µm

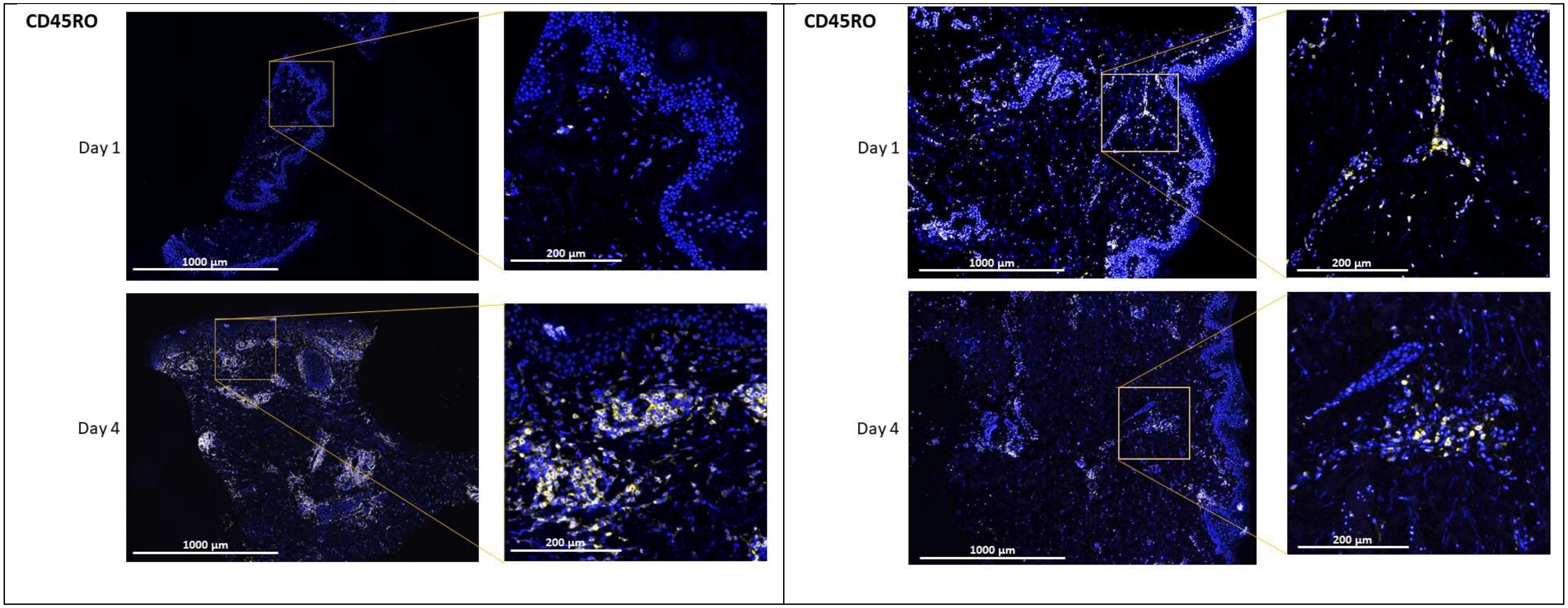
CD45RO
Day 1
Day 4
1000 µm
200 µm
1000 µm
200 µm
CD45RO
Day 1
Day 4
1000 µm
200 µm
1000 µm
200 µm

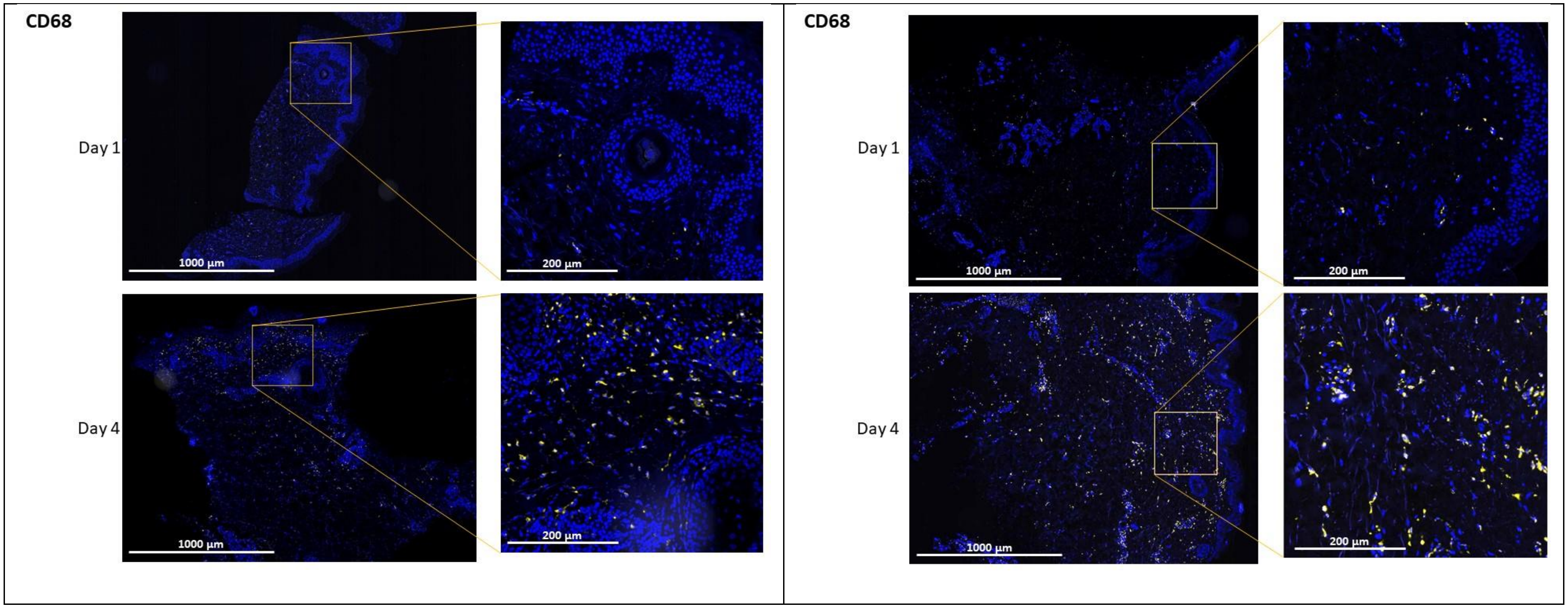
CD68
Day 1
Day 4
1000 µm
200 µm
1000 µm
200 µm
CD68
Day 1
Day 4
1000 µm
200 µm
1000 µm
200 µm

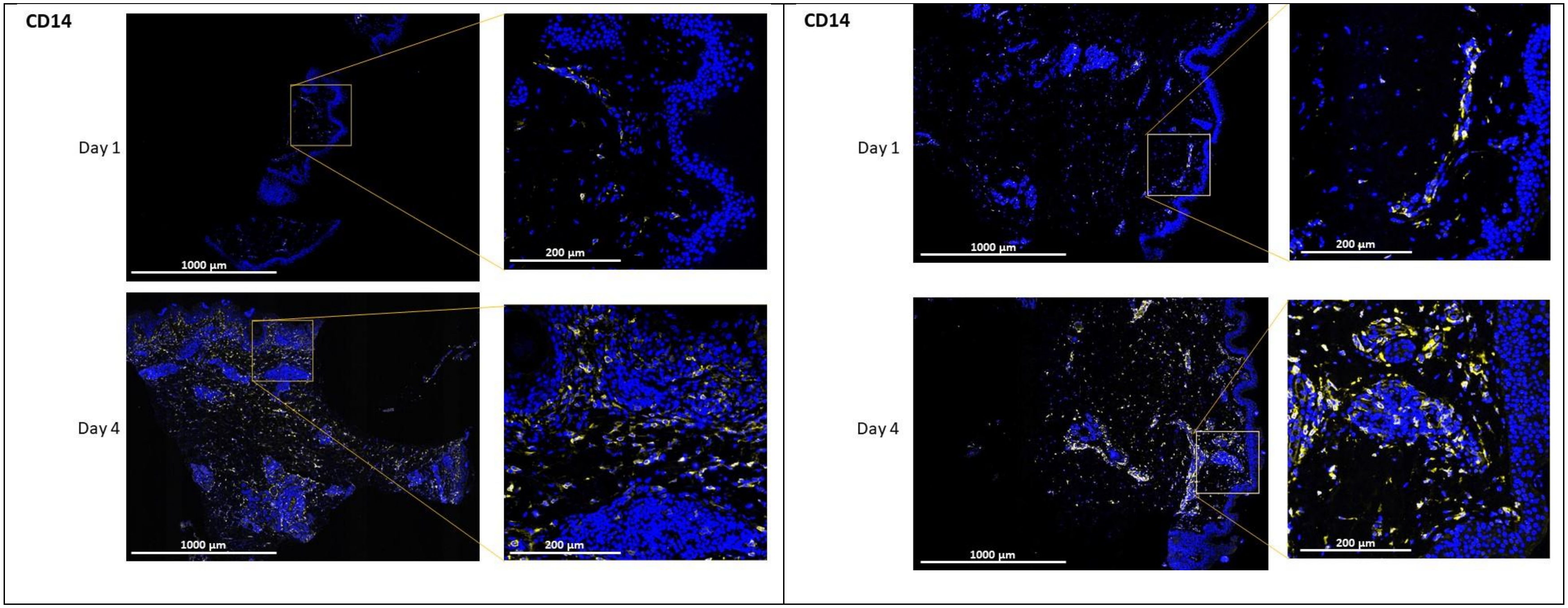
CD14
Day 1
1000 µm
200 µm
Day 4
1000 µm
200 µm
CD14
Day 1
1000 µm
200 µm
Day 4
1000 µm
200 µm

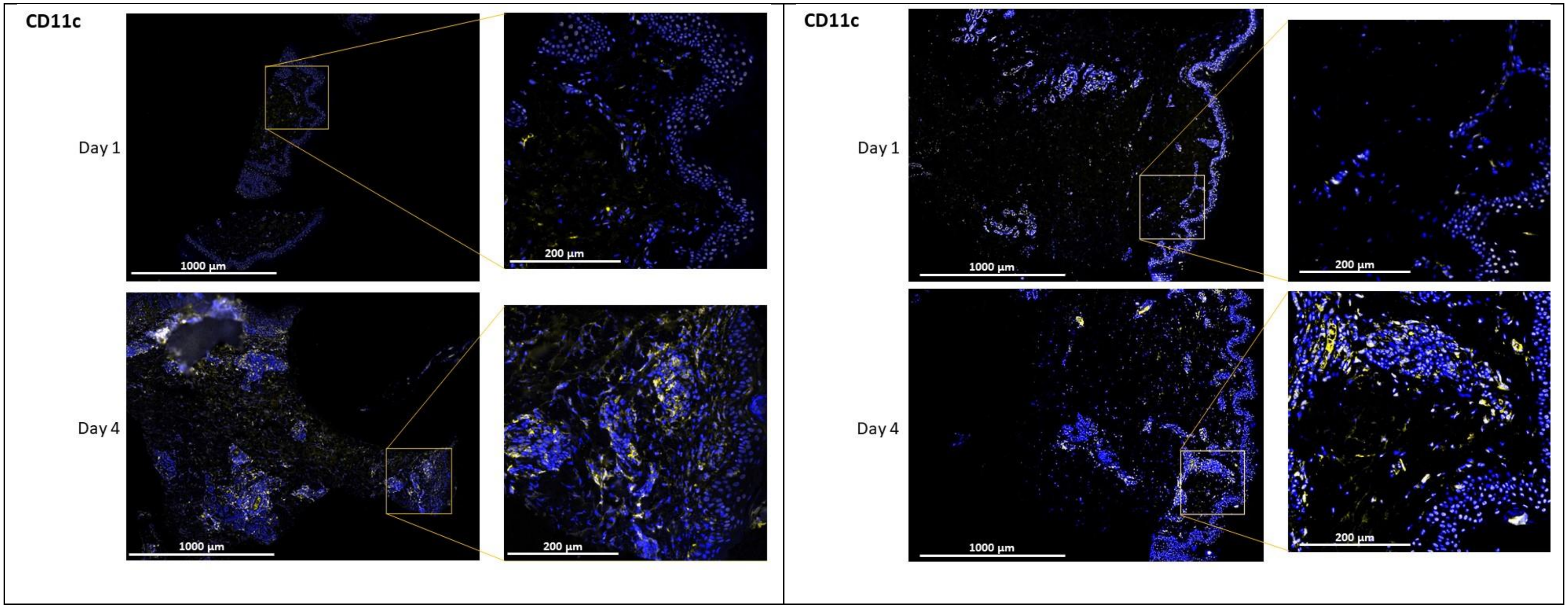
CD11c
Day 1
1000 µm
200 µm
Day 4
1000 µm
200 µm
CD11c
Day 1
1000 µm
200 µm
Day 4
1000 µm
200 µm

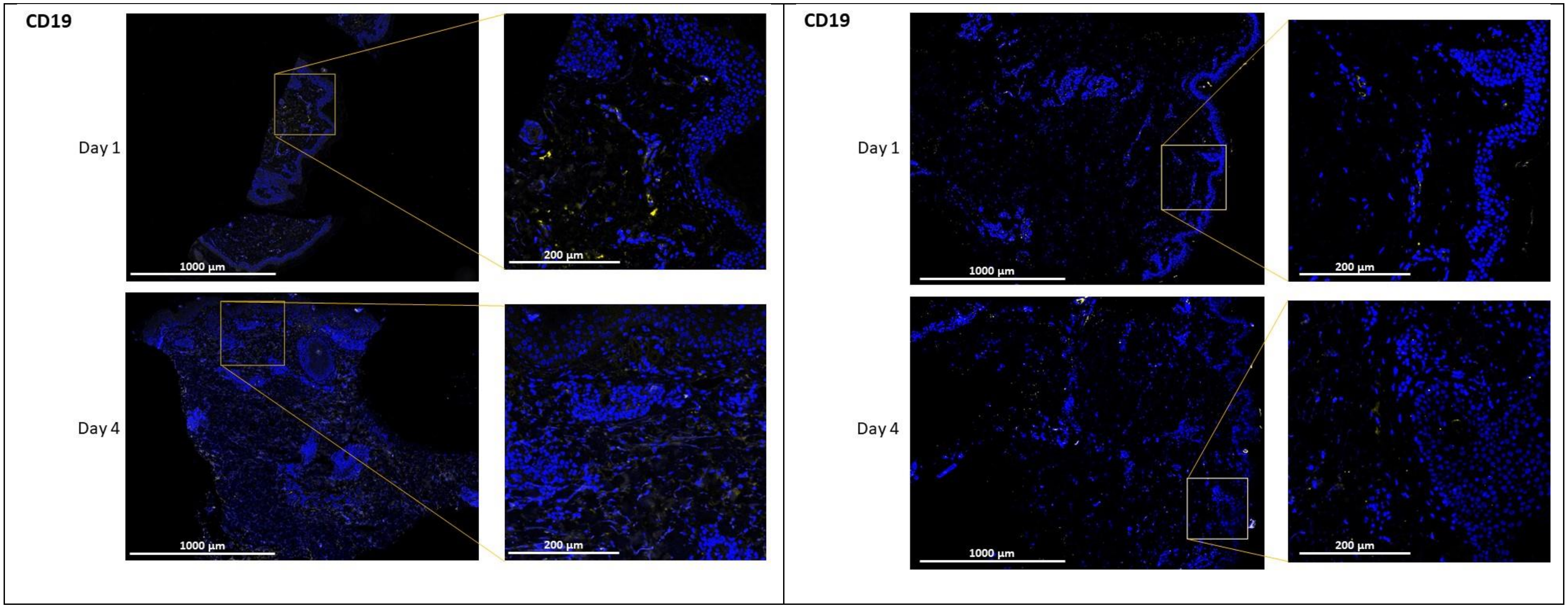
CD19
Day 1
1000 µm
200 µm
Day 4
1000 µm
200 µm
CD19
Day 1
1000 µm
200 µm
Day 4
1000 µm
200 µm

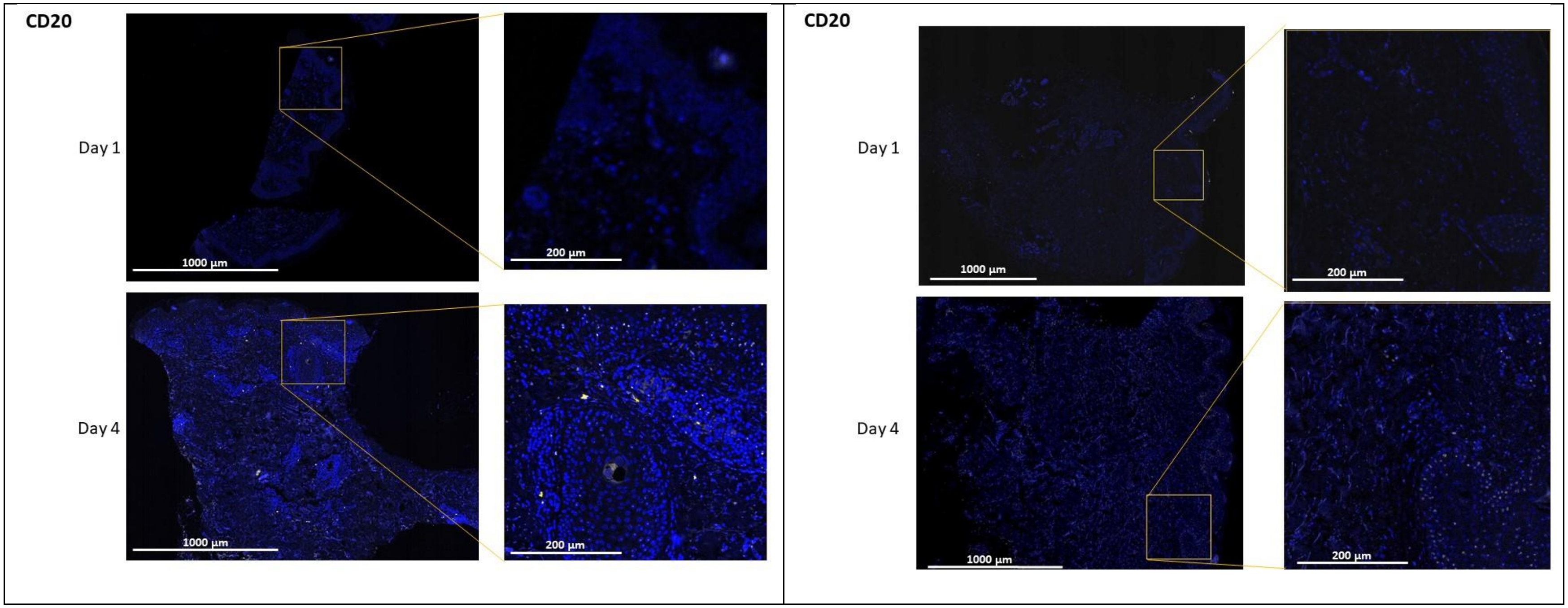
CD20
Day 1
1000 µm
200 µm
Day 4
1000 µm
200 µm
CD20
Day 1
1000 µm
200 µm
Day 4
1000 µm
200 µm

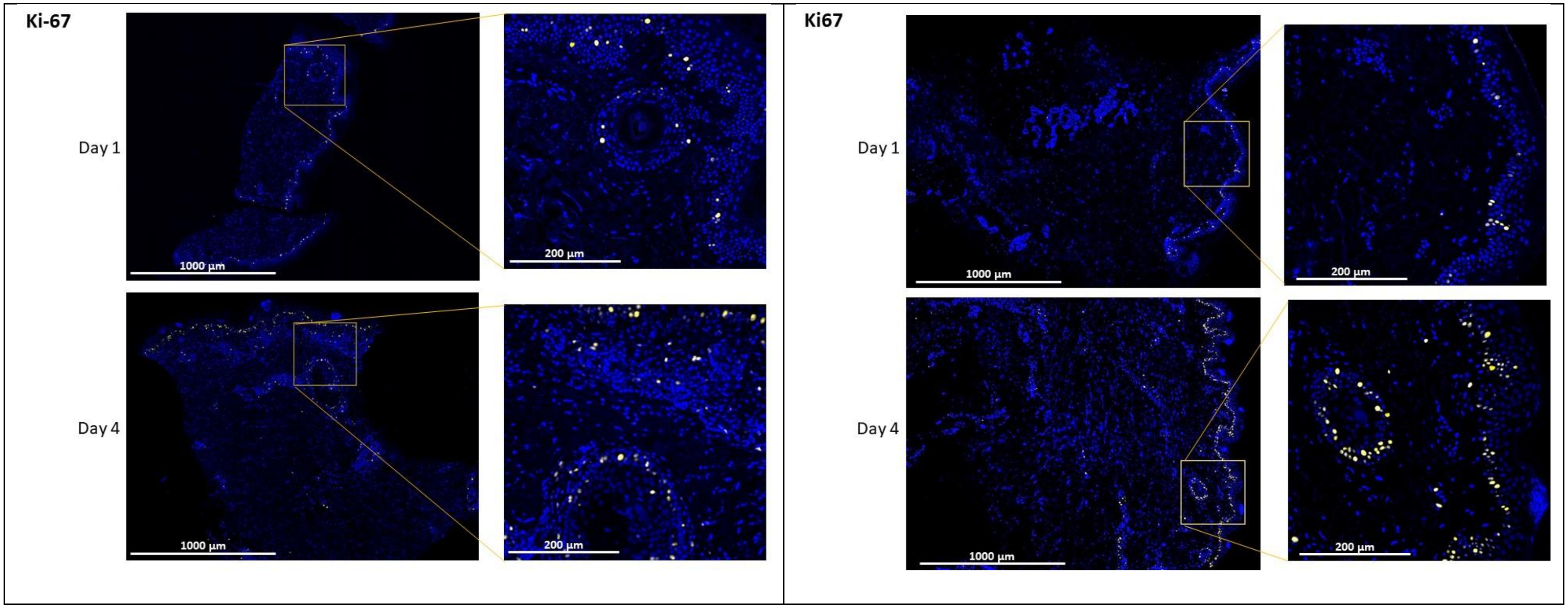

S4 Fig. Immunofluorescence slides from two representative subjects. Subjects received active/vaccine-coated HD-MAP (left) or placebo HD-MAP (right). Biopsies were taken on day 1 (pre) and 4 (post) HD-MAP application. Following fixation, paraffin-embedded 5 µm thick sections were subjected to immunofluorescent staining. DAPI (blue); AF555 (yellow) for specific cell surface markers.
