# Supplementary material for: Cellular responses at the application site of a high-density microarray patch delivering an influenza vaccine in a randomized, controlled phase I clinical trial
Source: PLoS One. 2021 Jul 30;16(7):e0255282. doi: 10.1371/journal.pone.0255282 (PMC8323919; doi:10.1371/journal.pone.0255282)
Supplement: S1 Table — (PDF) [file pone.0255282.s005.pdf]

S1 Table. Antibodies used for flow-cytometry analysis of biopsies.

Reagent/Stain	*Supplier (Cat#)*	*Final Dilution*
Zombie Aqua Live/Dead (BV510)	Biolegend (423101)	1:800
Human TruStain FcX	Biolegend (422302)	1:60
α-CD3 (BV786)	BD Biosciences (563800)	1:200
α-αβTCR (BV786)	BD Biosciences (563825)	1:100
α-CD103 (BV421)	BD Biosciences (563882)	1:100
α-CD68 (BV711)	BD Biosciences (565594)	1:100
α-CD11c (BV605)	BD Biosciences (563929)	1:50
α-CD8b (BB700)	BD Biosciences (745761)	1:800
α-HLA-DR (FITC)	BD Biosciences (555560)	1:50
α-CD45 (PE-CY5)	BD Biosciences (555484)	1:400
α-CD45RO (PE)	BD Biosciences (555493)	1:50
α-CD11b-Mac-1 (PE-CY7)	BD Biosciences (557743)	1:100
α-CD4 (AlexaFluor 700)	BD Biosciences (557882)	1:400
α-CD69 (APC)	BD Biosciences (555751)	1:50
α-CD19 (APC-CY7)	BD Biosciences (557791)	1:100
α-CD20 (APC-CY7)	BD Biosciences (335794)	1:100
