# Supplementary material for: Cellular responses at the application site of a high-density microarray patch delivering an influenza vaccine in a randomized, controlled phase I clinical trial
Source: PLoS One. 2021 Jul 30;16(7):e0255282. doi: 10.1371/journal.pone.0255282 (PMC8323919; doi:10.1371/journal.pone.0255282)
Supplement: S2 Table — (PDF) [file pone.0255282.s006.pdf]

S2 Table. Antibodies used for immunohistochemistry.

Antibody	***Supplier/Antigen Retrieval***	***Temperature***	***Secondary Antibody staining***		***Time (s)***
CD3	Diva	15’@105°C	1:100/VGY	anti-rabbit 555	45
CD4	Dako 9	10’@110°C	1:20/VGY	anti-mouse 555	120
CD8	Diva	5’@125°C	1:200/VGY	anti-mouse 555	60
CD11c	Diva	15’@105°C	1:100/DVG	anti-rabbit 555	60
CD14	Diva	15’@105°C	1:100/VGY	anti-rabbit 555	45
CD19	Dako 9	8’@120°C	1:100/VGY	anti-rabbit 555	45
CD20	Dako 6	5’@125°C	1:200/VGY	anti-mouse 555	45
CD45RO	Dako 9	10’@110°C	1:80/VGY	anti-mouse 555	60
CD68	Dako 6	5’@125°C	1:100/VGY	anti-mouse 555	45
Ki-67	Dako 6	5’@125°C	1:100/DVG	anti-mouse 555	45
HLA-DR	Dako 6	10’@110°C	1:60/DVG	anti-mouse 555	45
