# Supplementary material for: Cellular responses at the application site of a high-density microarray patch delivering an influenza vaccine in a randomized, controlled phase I clinical trial
Source: PLoS One. 2021 Jul 30;16(7):e0255282. doi: 10.1371/journal.pone.0255282 (PMC8323919; doi:10.1371/journal.pone.0255282)
Supplement: S1 Methods — (PDF) [file pone.0255282.s008.pdf]

S1 Methods

Flow cytometry, staining and analysis

Zombie Aqua Live/Dead stain diluted in DPBS was added to each FACS tube containing cells prior to samples being blocked with Human TruStain FcX (cat 422302, BioLegend, USA) on ice for 15 min prior to antibody staining with 14 markers (see Supplementary Table 2a) diluted in PBS/2% HI FCS/2mM EDTA for 30 min protected from light on ice. Cells were washed with PBS/2% HI FCS/2mM EDTA and before 20,000 counting beads (FlowCount Fluorospheres, Beckman Coulter) were added; all samples were made to a final volume of 400 µL with PBS/2% HI FCS/2mM EDTA for analysis. Analyses were performed on a BD LSR Fortessa Flow Cytometer in conjunction with BD FACS Diva Software. Compensation for all colours was completed using compensation beads and event exclusion threshold was set to 5,000 events. Data were analysed in FlowJo V10.4. 'Fluorescence Minus One' (FMO) controls were performed for each colour using PBMCs. Gating within channels was performed with reference to the FMOs plus minor adjustments to reflect known skin characteristics. In place of a lymphocyte gate, a >20,000K cut-off gate was used as back-gating showed that staining for markers of interest fell outside this gate (e.g. monocytes, granulocytes, neutrophils). The gating strategy is shown in S1 Fig.

Immunohistochemistry (immunofluorescence) – Preparation, imaging and analysis

Sections were stained either with Haematoxylin and Eosin (H&E) (Haematoxylin, Sigma-Aldrich, USA; Eosin Y, ProScitech, Australia), or for immunofluorescence (IF). Eleven primary antibodies and three secondary antibodies conjugated to AF555 were used (Supplementary table S2b). Antibody stained sections were co-stained with DAPI (Sigma-Aldrich D9542, 300 nM). Human tonsil tissue was used for internal, negative control slides (incubated without primary antibodies, instead of isotype controls). Two to four microscope slides/sections were imaged for each subject. Four paraffin-embedded H&E-stained tissue matrix arrays (TMAs) were scanned on the Olympus VS120 slide scanner using Olympus VS-ASW software (version 2.9), with a 20x air objective (Olympus). OlyVIA (version 2.6), Visiopharm (version 2017.2.4.3387), and Fiji (ImageJ, version 1.52H) software was used for analyses. IF slides were imaged on a Leica Biosystems Aperio ScanScope FL with an 8/10-bit monochrome TDI line-image capture camera and mercury light source. An Olympus lens (UPLSApo) UIS coupled with a 20x/0.75 PlanSApo objective (Olympus) acquired at 0.468 µm/pixel DAPI and AF555 sequentially through a 4040C filter set (nominally Cy3; Ex/Em 531/593 +/- 10 nm), using ScanScope software (version 102.0.0.33).

H&E stained images were visualised in OlyVIA, and analysed for tissue area, thickness and cell sizes in Visiopharm. Individual strata thicknesses were measured in Fiji and added together to obtain an approximate total tissue thickness. Due to the inability of counting cells directly within the Aperio Microscope system, cell numbers were quantified by converting approximate cell area sizes into cell counts. For cell quantification, all strata were measured as area and circumferences in Visiopharm, first as whole tissue area, followed by individual strata, and dividing the added area measurements of the respective colour (e g purple for cell nuclei) by the average cell size. The average size of keratinocytes was 38.73 ± 14.11 μm^2 whilst infiltrating cells measured approximately 17.13 ± 4.91 μm^2 based on at least n=25 measurements per subject/slide,

respectively. To quantify the approximate number of infiltrating cells into the dermis, the two haematoxylin measurements of the dermis area were added (all nuclei), and then divided by marker-specific averaged cell-size measurements (in μm^2) of infiltrating cells.
